# MRI radiomics based on deep learning automated segmentation to predict early recurrence of hepatocellular carcinoma

**DOI:** 10.1186/s13244-024-01679-8

**Published:** 2024-05-20

**Authors:** Hong Wei, Tianying Zheng, Xiaolan Zhang, Yuanan Wu, Yidi Chen, Chao Zheng, Difei Jiang, Botong Wu, Hua Guo, Hanyu Jiang, Bin Song

**Affiliations:** 1 Department of Radiology, Functional and Molecular Imaging Key Laboratory of Sichuan Province, West China Hospital, Sichuan University, Chengdu, Sichuan 610041 China; 2 Shukun Technology Co., Ltd, Beijing, 100102 China; 3 Big Data Research Center, University of Electronic Science and Technology of China, Chengdu, Sichuan 610000 China; 4 Center for Biomedical Imaging Research, Department of Biomedical Engineering, School of Medicine, Tsinghua University, Beijing, 100102 China; 5 Department of Radiology, Sanya People’s Hospital, Sanya, Hainan 572000 China

**Keywords:** Artificial intelligence, Carcinoma (hepatocellular), Recurrence, Magnetic resonance imaging, Machine learning

## Abstract

**Objectives:**

To investigate the utility of deep learning (DL) automated segmentation-based MRI radiomic features and clinical-radiological characteristics in predicting early recurrence after curative resection of single hepatocellular carcinoma (HCC).

**Methods:**

This single-center, retrospective study included consecutive patients with surgically proven HCC who underwent contrast-enhanced MRI before curative hepatectomy from December 2009 to December 2021. Using 3D U-net-based DL algorithms, automated segmentation of the liver and HCC was performed on six MRI sequences. Radiomic features were extracted from the tumor, tumor border extensions (5 mm, 10 mm, and 20 mm), and the liver. A hybrid model incorporating the optimal radiomic signature and preoperative clinical-radiological characteristics was constructed via Cox regression analyses for early recurrence. Model discrimination was characterized with C-index and time-dependent area under the receiver operating curve (tdAUC) and compared with the widely-adopted BCLC and CNLC staging systems.

**Results:**

Four hundred and thirty-four patients (median age, 52.0 years; 376 men) were included. Among all radiomic signatures, HCC *with* *5* *mm* *tumor* *border* *extension* *and* *liver* showed the optimal predictive performance (training set C-index, 0.696). By incorporating this radiomic signature, rim arterial phase hyperenhancement (APHE), and incomplete tumor “capsule,” a hybrid model demonstrated a validation set C-index of 0.706 and superior 2-year tdAUC (0.743) than both the BCLC (0.550; *p* < 0.001) and CNLC (0.635; *p* = 0.032) systems. This model stratified patients into two prognostically distinct risk strata (both datasets *p* < 0.001).

**Conclusion:**

A preoperative imaging model incorporating the DL automated segmentation-based radiomic signature with rim APHE and incomplete tumor “capsule” accurately predicted early postsurgical recurrence of a single HCC.

**Critical relevance statement:**

The DL automated segmentation-based MRI radiomic model with rim APHE and incomplete tumor “capsule” hold the potential to facilitate individualized risk estimation of postsurgical early recurrence in a single HCC.

**Key Points:**

- A hybrid model integrating MRI radiomic signature was constructed for early recurrence prediction of HCC.
- The hybrid model demonstrated superior 2-year AUC than the BCLC and CNLC systems.
- The model categorized the low-risk HCC group carried longer RFS.

**Graphical Abstract:**

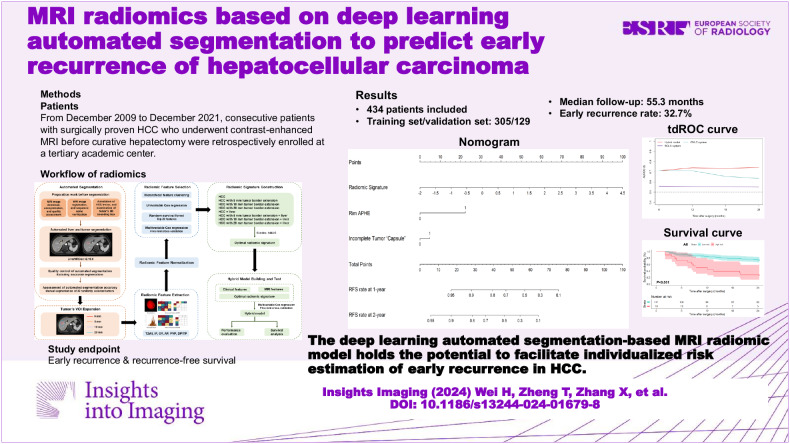

## Introduction

Hepatocellular carcinoma (HCC) is the sixth leading type of cancer and the third most fatal malignancy worldwide [1]. Surgical resection is recommended as the first-line treatment for early-stage HCC [2, 3]. However, even after curative-intent resection, tumor recurrence occurs in ~70% of patients [1, 2], whilst early recurrence within two years accounts for > 70% of recurrence [4, 5]. Tumor burden and aggressive characteristics, such as worse tumor differentiation, microvascular invasion (MVI), and satellite nodules, have been reported to be associated with early recurrence in HCC [4, 6–8]. Nonetheless, histopathological biomarkers had limited implications for clinical decision-making in the pretreatment context. Therefore, noninvasive estimation of early recurrence risk in HCC is crucial for individualized treatment.

Magnetic resonance imaging (MRI) is instrumental in the noninvasive diagnosis and management of HCC. Several semantic MR imaging features, such as rim arterial phase hyperenhancement (APHE), arterial phase peritumoral enhancement, and hepatobiliary phase (HBP) peritumoral hypointensity, have been associated with early recurrence of HCC [8, 9]. However, these semantic features are inadequate for prognostication due to limited predictive performances and suboptimal interobserver reproducibility.

Radiomics has emerged as a new radiological technique that enables the extraction of high-throughput quantitative image features beyond inspections of naked human eyes from standard-of-care medical images, providing important insights into cancer phenotypes and tumor microenvironments that are distinct and complementary to other clinical information [10]. Previous studies have shown good predictive accuracy of MRI radiomic analyses for HCC recurrence after surgery [6, 11–13]. However, these studies generally included limited sample sizes (e.g., 48–361 patients) and utilized manual or semiautomated segmentation, which are time-consuming, labor-intensive, operator-dependent, and subject to inter-rater variability. Fortunately, with recent advances in artificial intelligence (AI) deep learning (DL) algorithms, liver and HCC lesions can be segmented in an automated manner, which may improve both efficiency and reproducibility [14–16]. DL image segmentation models enable the fully automated detection of tumor margins for fast and reproducible HCC segmentation. Accurate segmentation of liver and tumors is a critical prerequisite for subsequent quantitative analysis and holds the huge potential to standardize and improve clinical management. Nevertheless, to our knowledge, there is currently limited evidence on the utility of automated segmentation-based MRI radiomic analyses for predicting postoperative early recurrence in HCC.

Therefore, using the DL-assisted automated segmentation technique, this study aimed to develop and validate a predictive model for early recurrence based on MRI radiomic features and clinical-radiological characteristics in patients with single early-stage HCC following curative resection.

## Materials and methods

This single-institution, retrospective study was approved by the institutional review board of West China Hospital of Sichuan University, with a waiver of the informed consent.

### Patients

**Fig. 1:**
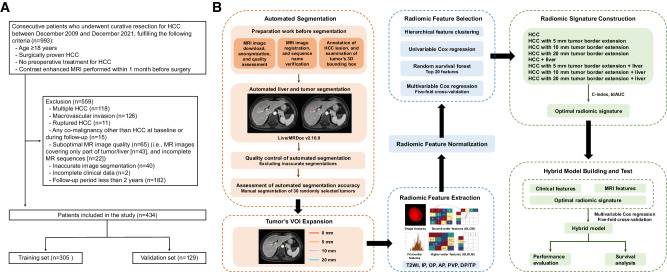
Flowcharts depicting (**A**) the recruitment of patients and (**B**) the workflow of radiomics. AP, arterial phase; C-index, concordance index; DP, delayed phase; OP, opposed phase; PVP, portal venous phase; tdAUC, time-dependent area under the receiver operating characteristic curve; TP, transitional phase; T2WI, T2-weighted imaging; VOI, volume of interest; 3D, three dimensional

Consecutive patients who received curative resection for HCC between December 2009 and December 2021 were retrospectively recruited. The inclusion criteria were: (a) age ≥ 18 years, (b) surgically proven HCC, (c) no preoperative treatment for HCC, and (d) contrast-enhanced MRI performed within 1 month before surgery. The exclusion criteria were: (a) multiple HCC, (b) macrovascular invasion, (c) ruptured HCC, (d) any co-malignancy other than HCC at baseline or during follow-up, (e) suboptimal MR image quality (i.e., MR images covering only part of tumor/liver, and incomplete MR sequences), (f) inaccurate image segmentation (detailed below), (g) incomplete clinical data (detailed below), and (h) follow-up period less than 2 years. Eligible patients were randomly assigned to training and validation sets at a ratio of 7:3 (Fig. 1A).

Baseline clinical, laboratory, and histopathological data were collected from the electronic medical records. Cirrhosis was diagnosed according to the Clinical Practice Guidelines [17]. Intraoperative ultrasound was routinely performed for each patient to detect small occult HCCs and guide the resection strategy.

Of note, 16.8% (73/434) of these patients have been reported in our prior work [18], where the primary focus was on the MRI features associated with HCC recurrence without radiomic model construction.

### MRI technique

MRI was performed with various 3.0-T or 1.5-T scanners. The choice of MRI contrast agents, either extracellular or hepatobiliary, was determined by the surgeons or multidisciplinary team. MRI systems and acquisition protocols are detailed in Supplementary Material 1 and Table S1.

### MRI evaluation

Two abdominal radiologists (H.Y.J. and H.W., with 8 and 5 years of experience in liver MRI, respectively) independently reviewed all deidentified MR images. They were informed of the HCC diagnosis but were blinded to other clinical, histopathological, and follow-up information. Discrepancies between the two readers were resolved by a senior abdominal radiologist with over 20 years of experience in liver MRI.

On a per-lesion basis, the following features were evaluated: (a) tumor size (cm), (b) enhancement pattern (typical vs atypical, with typical enhancement pattern referring to the presence of non-rim APHE coupled with nonperipheral “washout” [19]), (c) rim APHE, (d) corona enhancement, (e) nonsmooth tumor margin, (f) incomplete tumor “capsule,” (g) delayed central enhancement, (h) enhancing “capsule,” (i) intratumoral necrosis, (j) fat in mass, more than adjacent liver, (k) radiological cirrhosis, (l) diffuse fatty change, (m) diffuse iron overload, (n) splenomegaly, (o) ascites, (p) collateral circulation, (q) gastroesophageal varices, and (r) main portal vein diameter (cm). Definitions of the imaging features haven been described in our prior study [20].

### Radiomic analysis

#### Image acquisition, preprocessing, and automated segmentation

De-identified MR images were uploaded to a commercial visualization and analysis software (LiverMRDoc; version 2.10.0; Shukun Technology Co., Ltd).

Before automated segmentation, one radiologist (H.W.) inspected all MR images in terms of the sequence names, HCC lesions, and corresponding 3D bounding boxes (i.e., the automated lesion detection annotation) on the AI software platform. To ensure accurate localization of tumors, manual adjustment was conducted for 16 patients with inaccurate 3D bounding boxes (e.g., failing to detect HCC lesions or delineate the whole tumors).

Using 3D U-net-based DL algorithms as detailed in Supplementary Material 2 and Fig. S1, automated segmentation of liver and HCC lesions was conducted on each transverse section of T2-weighted imaging (T2WI), IP, opposed phase (OP), arterial phase (AP), portal venous phase (PVP), and delayed phase (DP; for MRI with extracellular contrast agent [ECA]) or translational phase (TP; for MRI with hepatobiliary contrast agent [HCA]) images.

**Fig. 2:**
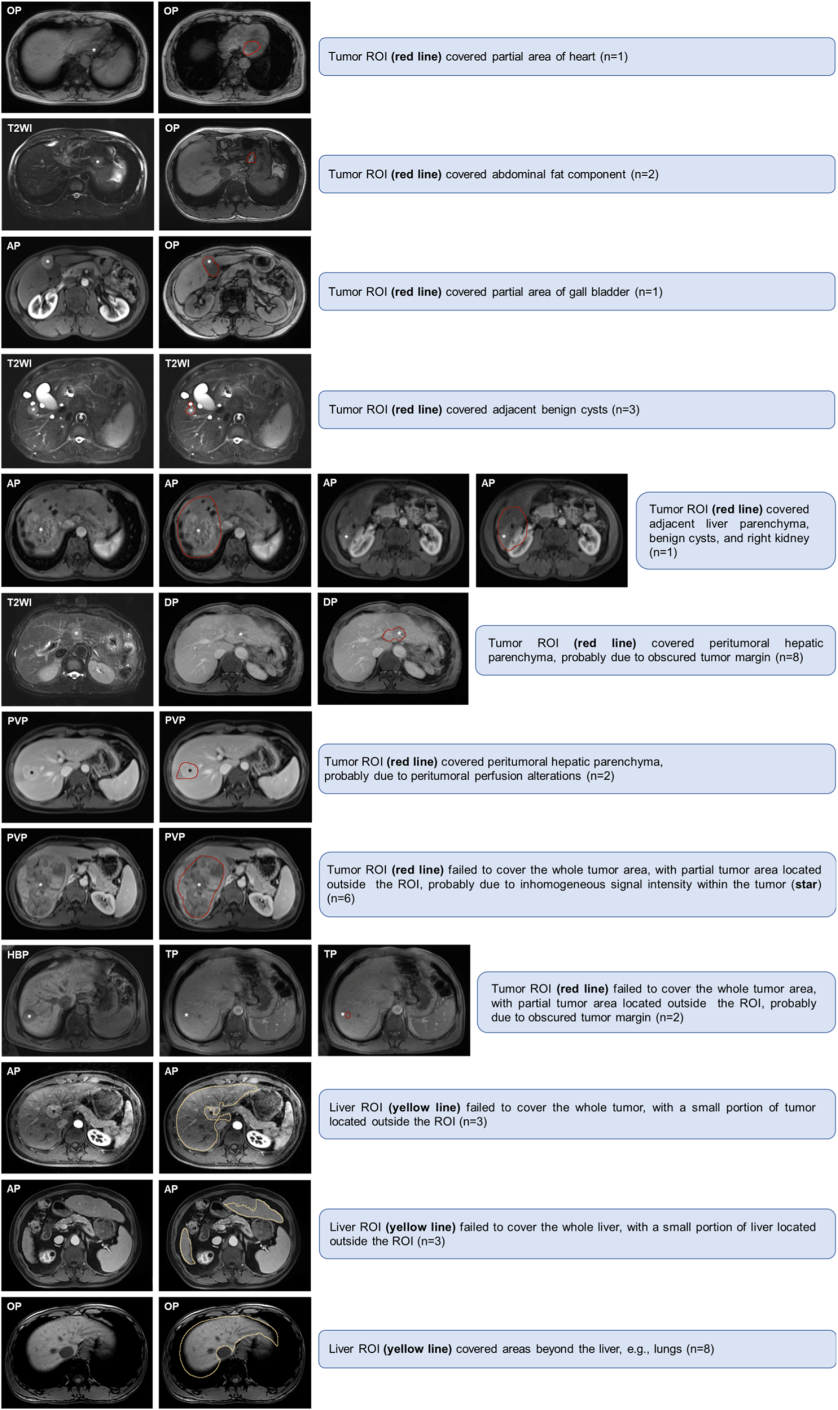
Examples of inaccurate image segmentations. AP, arterial phase; DP, delayed phase; OP, opposed phase; PVP, portal venous phase; ROI, region of interest; TP, transitional phase; T2WI, T2-weighted imaging

To implement the quality control, one radiologist (H.W.) visually inspected each segmented tumor and liver, and those (*n* = 40) with inaccurate tumor or liver segmentations on any above sequences were excluded from radiomic analyses. The exclusion criteria for inaccurate segmentation were (a) tumor region of interest (ROI) covered nontumoral areas (e.g., liver parenchyma, benign cysts, adjacent organs or tissues) (*n* = 18), (b) tumor ROI failed to cover the whole tumor areas (*n* = 8), (c) liver ROI failed to cover the whole tumor or liver areas (*n* = 6), and (d) liver ROI covered areas beyond the liver (*n* = 8). Examples of inaccurate image segmentations are presented in Fig. 2. Manual adjustment was not considered because the study aimed to examine the prognostic utility of this automated technique.

To assess the accuracy of automated DL segmentation, one radiologist (T.Y.Z., with 5 years of experience in liver MRI) who was unknown to the automated segmentation results manually segmented 30 randomly chosen HCC lesions using ITK-SNAP (version 3.8.0; www.itksnap.org).

**Fig. 3:**
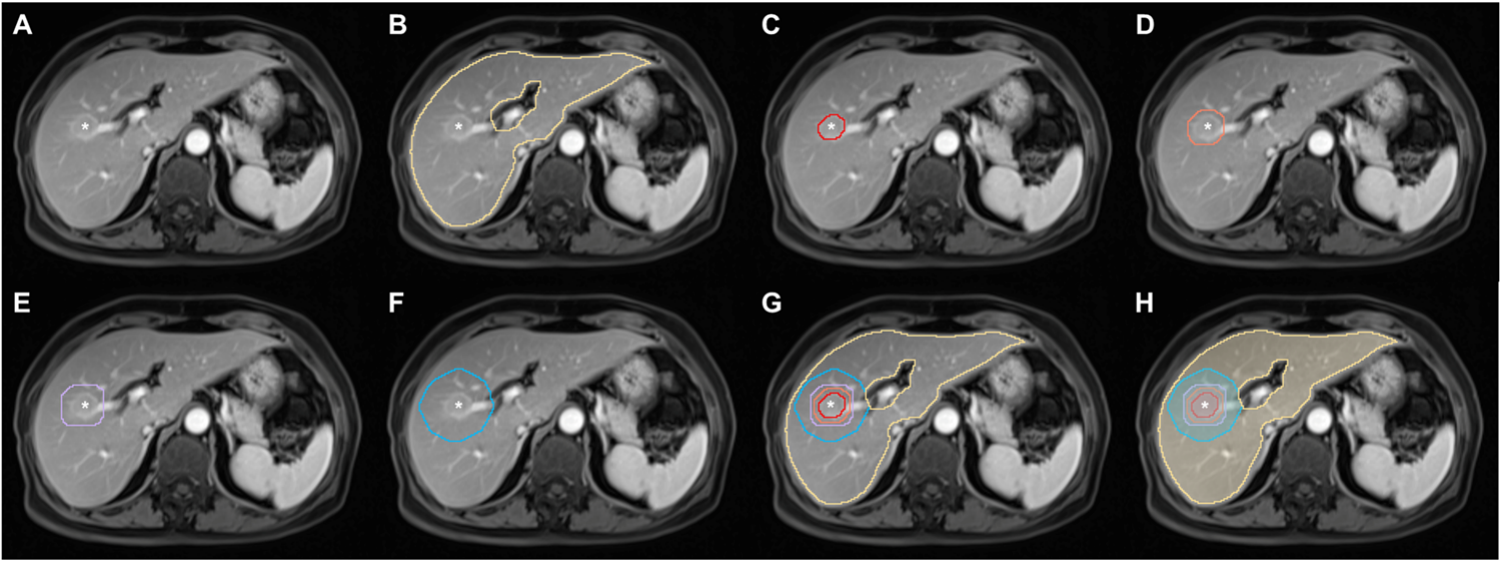
An example of automated segmentation. Axial MRI scans in a 71-year-old woman demonstrate a 2.1 cm HCC (*) in segments V and VIII of the liver on (**A**) portal venous phase image. Automated (**B**) liver segmentation (yellow lines), (**C**) tumor segmentation (read line), tumor border extensions with (**D**) 5 mm (orange line), (**E**) 10 mm (purple line), and (**F**) 20 mm (blue line) to create (**G**, **H**) corresponding segmentation masks

To extract radiomic features of peritumoral areas, the tumor’s 3D mask was expanded radially outwards by 5, 10, and 20 mm on each sequence using a medical research platform (UltraScholar, Version 2.0, Shukun Technology Co., Ltd, https://medresearch.shukun.net/). Accordingly, five types of VOIs were created: (a) tumor VOI, defined as the VOI covering HCC lesion; (b) three extended tumor VOIs, defined as the tumor VOI with automated extension of tumor boundaries by 5, 10, and 20 mm, respectively; and (c) liver VOI, defined as the VOI covering nontumoral liver parenchyma (Fig. 3).

#### Radiomic feature extraction

The radiomic workflow is illustrated in Fig. 1B. Detailed methods of radiomic analyses are shown in Supplementary Material 3. MR signal intensity normalization and radiomic feature extraction were performed with the PyRadiomics package (version 3.0.1; https://pyradiomics.readthedocs.io/en/v3.0.1/).

A total of 1688 features were extracted from each VOI in one sequence. Radiomic features were extracted for VOIs of tumor, tumor border extensions (5, 10, and 20 mm), and the liver, respectively.

#### Radiomic feature normalization and abnormal feature exclusion

Radiomic feature normalization, abnormal feature exclusion, feature selection, and radiomic signature construction were performed with R software (version 4.3.1; The R Foundation for Statistical Computing).

Values of extracted radiomic features on the training set were normalized with z scores; the means and standard deviations derived from the training set were applied to the feature normalization of the validation set. Abnormal features with a variance of 0 were excluded from further analyses.

#### Feature selection

After normalization and excluding abnormal features, we followed a four-step procedure to reduce dimensions and select robust radiomic features on the training set. First, intervariable collinearity was estimated by Spearman correlation analysis. For radiomic features with a Spearman’s rank correlation coefficient > 0.8, hierarchical feature clustering was performed to remove redundancy. Subsequently, univariable Cox regression analysis was performed to identify significant radiomic features associated with early recurrence. Features with a *p* < 0.01 were kept for further analyses. Next, random survival forest (RSF) was used to select the top 20 features. Finally, radiomic signatures were constructed by the multivariable Cox regression analysis using a backward elimination approach with five-fold cross-validation.

#### Radiomic signature development and validation

Eight groups of radiomic signatures were built based on different combinations of radiomic features extracted from tumor, tumor border extensions, and the liver, including (a) HCC, (b) HCC with 5 mm tumor border extension, (c) HCC with 10 mm tumor border extension, (d) HCC with 20 mm tumor border extension, (e) HCC and liver, (f) HCC with 5 mm tumor border extension and liver, (g) HCC with 10 mm tumor border extension and liver, and (h) HCC with 20 mm tumor border extension and liver. The optimal radiomic signature that exhibited the highest performance was selected for building the hybrid model (detailed below).

### Patient follow-up

Postoperative follow-up consisted of serum alpha-fetoprotein (AFP) level, liver biochemistry, and contrast-enhanced imaging examinations (i.e., ultrasound, computed tomography, or MRI) performed 1 month after surgery, every 3 months in the first 2 years, and every 6 months thereafter. Patients were followed up until death or the end date of this study (May 1, 2022). Early recurrence was defined as tumor recurrence within 2 years after surgery. Recurrence-free survival (RFS) was defined as the time interval from surgery to the first documented tumor recurrence or death.

### Statistical analysis

Continuous variables were compared by Student’s *t* validation or Mann-Whitney *U* validation, whereas categorical variables were compared by chi-squared validation or Fisher’s exact validation, as appropriate. Interobserver agreement of MRI findings was measured with Cohen’s κ coefficient for binary features and intraclass correlation coefficient for continuous variables. The consistency between automated (A) and manual (M) segmentations was evaluated by calculating the Dice similarity coefficient (DSC), which was defined as DSC = 2 × ( | A | ∩ | M | )/( | A | ∪ | M | ) [21]. DSC is a widely used statistical metric that measures the proportion of overlapping pixels between two sets of image segmentations [22].

#### Model development and validation

Using the training set, a hybrid model was built by incorporating the optimal radiomic signature and clinical-radiological variables available before surgery. While controlling for age and sex, univariable Cox regression analysis was performed to identify significant predictors of early recurrence. The multicollinearity of variables was estimated by a variance inflation factor (VIF). For variables with VIF > 2, those with the largest absolute value of β coefficients were selected for further analyses. Variables with *p* < 0.1 in the univariable analysis following the above steps were entered into the multivariable Cox model; the final model was formulated by a backward elimination approach and the Akaike information criterion (AIC) with five-fold cross-validation.

Model discrimination and calibration were evaluated by the Harrell’s C-index [23] and calibration plot [24], respectively. The time-dependent receiver operating characteristic (tdROC) curve was used to estimate the prognostic accuracy at different time points [25]. The decision curve was plotted to measure the clinical utility of the model [26].

The hybrid model performance was compared with the widely used Barcelona Clinic Liver Cancer (BCLC) staging system [3] and the Chinese National Liver Cancer (CNLC) staging system [27].

#### Survival analysis

To stratify patients into high and low-risk groups for early recurrence, the optimal threshold for the proposed hybrid model was determined by X-tile software (version 3.6.1; Yale University School of Medicine). RFS was estimated by the Kaplan-Meier method and compared with the log-rank validation. Subgroup analyses were performed according to histological differentiation and MVI statuses, which were known pathological risk factors related to early recurrence of HCC [4, 7].

Statistical analyses were performed with R software (version 4.3.1; The R Foundation for Statistical Computing) or SPSS software (version 26.0; SPSS Inc.). Two-tailed *p* < 0.05 was considered statistically significant.

## Results

### Patient characteristics

A total of 434 patients (median age, 52.0 years; interquartile range [IQR], 45.0–60.0 years; 376 men) were included, with 305 (70.3%) and 129 (29.7%) patients on the training and validation sets, respectively. During a median follow-up period of 55.3 months (IQR, 39.0–79.8 months), early recurrence occurred in 32.7% (142/434) of patients.

The validation set had a higher proportion of patients with CNLC Ib stage (31.8% vs. 22.0%; *p* = 0.031) and more frequent diffuse iron overload (27.9% vs. 19.0%; *p* = 0.040) compared to the training set, whereas no differences were found in other clinical-radiological-pathological characteristics and follow-up data between the two datasets (*p* range, 0.223–0.963).

Patient characteristics are summarized in Table 1. MRI features and interobserver agreement are shown in Table 2.

**Table 1:** Patient characteristics

Characteristic	Whole cohort	Training set	Validation set	*p* value
	(*n* = 434)	(*n* = 305)	(*n* = 129)	
Age (y)^a^	52.0 (45.0–60.0)	51.0 (45.0–60.0)	53.0 (45.0–60.0)	0.736
Sex				0.587
Women	58 (13.4)	39 (12.8)	19 (14.7)	
Men	376 (86.6)	266 (87.2)	110 (85.3)	
Cause of liver disease				0.303
HBV	414 (95.4)	293 (96.1)	121 (93.8)	
Non-HBV	20 (4.6)	12 (3.9)	8 (6.2)	
Cirrhosis				0.430
Absent	201 (46.3)	145 (47.5)	56 (43.4)	
Present	233 (53.7)	160 (52.5)	73 (56.6)	
Child-Pugh class				0.586
A	430 (99.1)	303 (99.3)	127 (98.4)	
B	4 (0.9)	2 (0.7)	2 (1.6)	
ALBI grade				0.866
1	352 (81.1)	248 (81.3)	104 (80.6)	
2	82 (18.9)	57 (18.7)	25 (19.4)	
BCLC stage				0.327
0	83 (19.1)	62 (20.3)	21 (16.3)	
A	351 (80.9)	243 (79.7)	108 (83.7)	
CNLC stage				**0.031**
Ia	326 (75.1)	238 (78.0)	88 (68.2)	
Ib	108 (24.9)	67 (22.0)	41 (31.8)	
Contrast agent type of MRI				0.315
ECA	340 (78.3)	235 (77.0)	105 (81.4)	
HCA	94 (21.7)	70 (23.0)	24 (18.6)	
Laboratory index				
AST (IU/L)^a^	31.5 (25.0–42.0)	31.0 (25.0–42.0)	32.0 (26.0–42.0)	0.352
ALT (IU/L)^a^	35.0 (24.0–49.8)	34.0 (24.0–47.0)	36.0 (24.0–54.0)	0.310
TBIL (umol/L)^a^	13.6 (9.7–17.5)	13.6 (9.6–17.4)	13.4 (9.9–18.2)	0.630
ALB (g/L)^b^	43.0 ± 4.3	43.0 ± 4.1	43.1 ± 4.6	0.860
PLT (×10^9/L)^a^	125.5 (89.0–166.0)	124.0 (87.0–167.0)	135.0 (90.0–166.0)	0.496
PT (S)^a^	11.9 (11.3–12.6)	11.9 (11.3–12.6)	11.9 (11.4–12.6)	0.829
INR^a^	1.0 (1.0–1.1)	1.0 (1.0–1.1)	1.0 (1.0–1.1)	0.432
GGT (IU/L)^a^	45.0 (28.0–77.0)	44.0 (28.0–71.0)	46.0 (28.0–87.0)	0.223
AFP (ng/mL)				0.137
≤ 400	333 (76.7)	240 (78.7)	93 (72.1)	
> 400	101 (23.3)	65 (21.3)	36 (27.9)	
Histopathological characteristics				
Tumor differentiation^c^				0.805
Well or Moderate	291 (67.5)	205 (67.9)	86 (66.7)	
Poor	140 (32.5)	97 (32.1)	43 (33.3)	
MVI^c^				0.371
Absent	115 (53.0)	77 (51.0)	38 (57.6)	
Present	102 (47.0)	74 (49.0)	28 (42.4)	
Follow-up period^a^	55.3 (39.0, 79.8)	54.6 (39.0, 77.4)	57.8 (39.0, 80.8)	0.764
Early recurrence rate, %	142 (32.7)	100 (32.8)	42 (32.6)	0.963
RFS Rate at 24-month, %^d^	67.3 (62.8, 71.8)	67.2 (61.9, 72.5)	67.4 (59.4, 75.4)	0.945

Statistically significant *p* values are bold

Unless indicated otherwise, data are the number of patients, with percentages in parentheses

^a^ Data are medians, with IRs in parentheses

^b^ Data are means ± standard deviations

^c^ There were 3 missing data for tumor differentiation and 217 missing data for MVI in the whole cohort

^d^ Numbers in parentheses are 95% confidence intervals (CI)

*ALB* albumin, *ALBI* albumin-bilirubin, *ALT* alanine aminotransferase, *AST* aspartate aminotransferase, *ECA* extracellular contrast agent, *GGT* gamma-glutamyl transferase, *HCA* hepatobiliary contrast agent, *HBV* hepatitis B virus, *INR* international normalized ratio, *PLT* platelet, *PT* prothrombin time, *TBIL* total bilirubin

**Table 2:** MRI characteristics and interobserver agreement

MRI characteristic	Whole cohort	Training set	Validation set	*p* value	Interobserver agreement^a^
	(*n* = 434)	(*n* = 305)	(*n* = 129)		
Tumor size (cm)^b^	3.4 (2.3–5.0)	3.3 (2.3–4.8)	3.6 (2.3–5.9)	0.281	0.985 (0.982, 0.988)
Enhancement pattern				0.571	0.526 (0.438, 0.613)
Typical	308 (71.0)	214 (70.2)	94 (72.9)		
Atypical	126 (29.0)	91 (29.8)	35 (27.1)		
Rim APHE				0.574	0.642 (0.497, 0.787)
Absent	408 (94.0)	288 (94.4)	120 (93.0)		
Present	26 (6.0)	17 (5.6)	9 (7.0)		
Corona enhancement				0.603	0.438 (0.351, 0.524)
Absent	261 (60.1)	181 (59.3)	80 (62.0)		
Present	173 (39.9)	124 (40.7)	49 (38.0)		
Nonsmooth tumor margin				0.915	0.496 (0.411, 0.581)
Absent	133 (30.6)	93 (30.5)	40 (31.0)		
Present	301 (69.4)	212 (69.5)	89 (69.0)		
Incomplete tumor “capsule”				0.703	0.318 (0.22, 0.415)
Absent	129 (29.7)	89 (29.2)	40 (31.0)		
Present	305 (70.3)	216 (70.8)	89 (69.0)		
Delayed central enhancement				0.897	0.288 (0.055, 0.522)
Absent	425 (97.9)	298 (97.7)	127 (98.4)		
Present	9 (2.1)	7 (2.3)	2 (1.6)		
Enhancing “capsule”				0.493	0.346 (0.222, 0.47)
Absent	40 (9.2)	30 (9.8)	10 (7.8)		
Present	394 (90.8)	275 (90.2)	119 (92.2)		
Intratumoral necrosis				0.232	0.705 (0.635, 0.776)
Absent	284 (65.4)	205 (67.2)	79 (61.2)		
Present	150 (34.6)	100 (32.8)	50 (38.8)		
Fat in mass, more than adjacent liver				0.284	0.416 (0.329, 0.502)
Absent	269 (62.0)	194 (63.6)	75 (58.1)		
Present	165 (38.0)	111 (36.4)	54 (41.9)		
Radiological cirrhosis				0.477	0.628 (0.552, 0.703)
Absent	159 (36.6)	115 (37.7)	44 (34.1)		
Present	275 (63.4)	190 (62.3)	85 (65.9)		
Diffuse fatty change				0.749	0.596 (0.455, 0.737)
Absent	401 (92.4)	281 (92.1)	120 (93.0)		
Present	33 (7.6)	24 (7.9)	9 (7.0)		
Diffuse iron overload				**0.040**	0.475 (0.37, 0.581)
Absent	340 (78.3)	247 (81.0)	93 (72.1)		
Present	94 (21.7)	58 (19.0)	36 (27.9)		
Splenomegaly				0.718	0.61 (0.537, 0.683)
Absent	223 (51.4)	155 (50.8)	68 (52.7)		
Present	211 (48.6)	150 (49.2)	61 (47.3)		
Ascites				0.303	0.401 (0.232, 0.57)
Absent	414 (95.4)	293 (96.1)	121 (93.8)		
Present	20 (4.6)	12 (3.9)	8 (6.2)		
Collateral circulation				0.865	0.406 (0.329, 0.484)
Absent	181 (41.7)	128 (42.0)	53 (41.1)		
Present	253 (58.3)	177 (58.0)	76 (58.9)		
Gastroesophageal varices				0.839	0.327 (0.251, 0.403)
Absent	195 (44.9)	138 (45.2)	57 (44.2)		
Present	239 (55.1)	167 (54.8)	72 (55.8)		
Main portal vein diameter (cm)^b^	1.5 (1.3–1.6)	1.5 (1.3–1.6)	1.5 (1.3–1.6)	0.792	0.677 (0.457, 0.794)
Radiomic signature^b^	−0.08 (−0.47–0.40)	−0.11 (−0.44–0.33)	0.03 (−0.48–0.51)	0.381	…

Statistically significant *p* values are bold

Unless indicated otherwise, data are the number of patients, with percentages in parentheses

^a^ Data are ICC for continuous variables and Cohen’s κ coefficient for binary variables, with 95% confidence intervals in parentheses. Interobserver agreement was assessed by the ICC or Cohen’s κ coefficient as follows: 0.01–0.20, slight agreement; 0.21–0.40, fair agreement; 0.41–0.60, moderate agreement; 0.61–0.80, substantial agreement; and 0.81–1.00, almost perfect agreement

^b^ Data are medians, with IRs in parentheses

*ICC* intraclass correlation coefficient

### Evaluation of automated segmentation accuracy

DSCs for each sequence are detailed in Table S2. For 30 randomly selected HCCs (median size, 4.8 cm; IQR, 3.5–8.4 cm), the mean DSC between automated and manual tumor segmentations was 0.84 ± 0.13 (median, 0.88; IQR, 0.82–0.92) in all sequences.

### Construction of radiomic signatures on the training set

The number of radiomic features in each step of feature selection on the training set is presented in Table S3. Based on the top 20 features determined by RSF, eight radiomic signatures for predicting early recurrence were constructed by multivariable Cox regression analyses (Table S4). Of these, the best performing radiomic signature for early recurrence was HCC with 5 mm tumor border extension and liver, which demonstrated a C-index of 0.696 (95%CI: 0.645, 0.746) on the training set.

There was no evidence of a difference in the C-index of the radiomic signature between MRI with extracellular contrast agent and hepatobiliary contrast agent subgroups on both training (0.673 [95%CI: 0.610, 0. 736] vs 0.743 [95%CI: 0.658, 0.828]; *p* = 0.210) and test (0.700 [95%CI: 0.598, 0.801] vs 0.692 [95%CI: 0.577, 0.808]; *p* = 0.934) sets.

### Construction and validation of the hybrid model on the training and validation sets

**Fig. 4:**
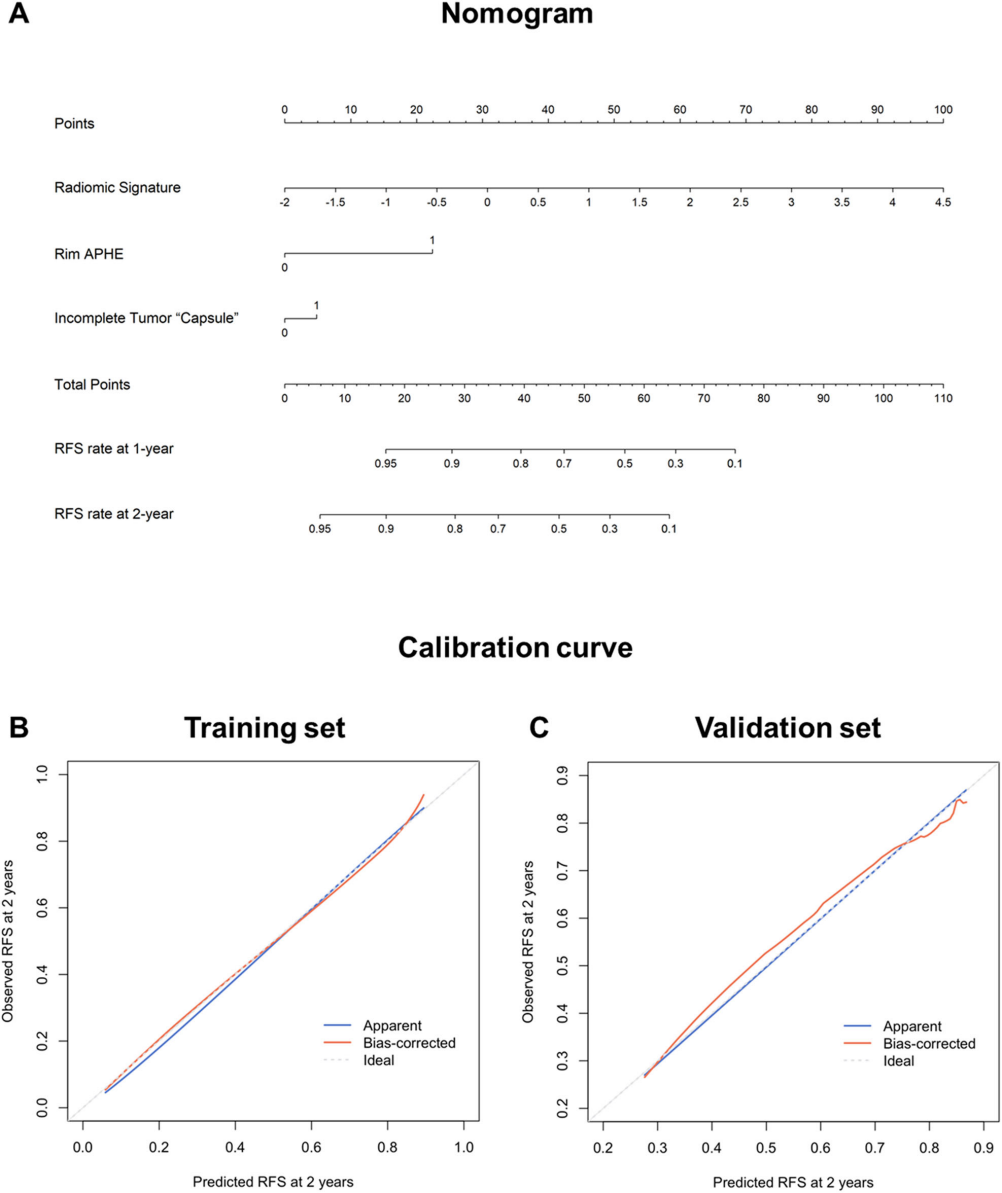
**A** The hybrid model-based nomogram to predict early recurrence of single HCC after surgical resection. Calibration curves of the hybrid model on the (**B**) training and (**C**) validation sets, respectively

The univariable analysis identified nine variables as potential predictors for early recurrence on the training set (*p* range, < 0.001–0.061). On subsequent multivariable analysis, rim APHE (hazard ratio [HR] = 4.315; 95%CI: 2.384, 7.810; *p* < 0.001), radiomic signature (HR = 2.728; 95%CI: 2.178, 3.417; *p* < 0.001) and incomplete tumor “capsule” (HR = 1.370; 95%CI: 0.831, 2.258; *p* = 0.217) were included in the Cox model (Table 3). A hybrid model that incorporated the above predictors were constructed for predicting early recurrence and illustrated as a nomogram to provide individualized risk estimates (Fig. 4A).

**Table 3:** Predictors for early recurrence based on cox regression analyses on the training set (*n* = 305)

Variable	Univariable analysis		Multivariable analysis	
	HR	*p* value	HR	*p* value
Age (> 50 y)	0.851 (0.575, 1.262)	0.423	…	…
Sex (male)	0.729 (0.420, 1.263)	0.259	…	…
Cause of liver disease (non-HBV)	1.065 (0.392, 2.897)	0.901	…	…
Cirrhosis	1.072 (0.722, 1.591)	0.731	…	…
ALBI grade (2)	0.865 (0.513, 1.459)	0.587	…	…
AST (> 40 IU/L)	1.140 (0.740, 1.757)	0.552	…	…
ALT (> 50 IU/L)	0.648 (0.383, 1.094)	0.105	…	…
TBIL (> 19 umol/L)	0.623 (0.353, 1.098)	0.102	…	…
ALB (< 40 g/L)	0.894 (0.551, 1.451)	0.651	…	…
PLT (< 100 ×10^9^/L)	0.970 (0.638, 1.474)	0.885	…	…
PT (> 13 S)	0.557 (0.270, 1.147)	0.112	…	…
INR (> 1.1)	0.687 (0.412, 1.144)	0.149	…	…
GGT (> 60 IU/L)	1.570 (1.043, 2.364)	**0.031**	…	…
AFP (> 400 ng/mL)	1.549 (0.987, 2.432)	**0.057**	…	…
BCLC stage (A)	1.550 (0.904, 2.656)	0.111	…	…
Tumor size (> 5 cm)	2.227 (1.460, 3.398)	**< 0.001**	…	…
Enhancement pattern (atypical)	0.880 (0.566, 1.368)	0.570	…	…
Rim APHE	4.670 (2.585, 8.435)	**< 0.001**	4.315 (2.384, 7.810)	< 0.001
Corona enhancement	1.599 (1.077, 2.373)	**0.020**	…	…
Nonsmooth tumor margin	1.555 (0.981, 2.466)	**0.061**	…	…
Incomplete tumor “capsule”	1.837 (1.123, 3.005)	**0.016**	1.370 (0.831, 2.258)	0.217
Delayed central enhancement	1.489 (0.469, 4,725)	0.500		
Enhancing “capsule”	0.716 (0.389, 1.318)	0.284		
Intratumoral necrosis	1.694 (1.136, 2.527)	**0.010**		
Fat in mass, more than adjacent liver	1.106 (0.738, 1.657)	0.626		
Radiological cirrhosis	0.914 (0.611, 1.367)	0.661	…	…
Diffuse fatty change	0.751 (0.327, 1.723)	0.498	…	…
Diffuse iron overload	1.011 (0.612, 1.669)	0.966	…	…
Splenomegaly	0.932 (0.629, 1.381)	0.725	…	…
Ascites	1.487 (0.594, 3.720)	0.397	…	…
Collateral circulation	1.044 (0.700, 1.556)	0.833	…	…
Gastroesophageal varices	1.183 (0.793, 1.765)	0.411	…	…
Main portal vein diameter (> 1.3 cm)	1.491 (0.915, 2.429)	0.109	…	…
Radiomic signature	2.773 (2.225, 3.456)	**< 0.001**	2.728 (2.178, 3.417)	< 0.001

Statistically significant *p* values are bold

The hybrid model exhibited a C-index of 0.727 (95%CI: 0.676, 0.777) on the training set and 0.706 (95%CI: 0.630, 0.783) on the validation set. There was no evidence of a difference in the C-index of the hybrid model between MRI with extracellular contrast agent and hepatobiliary contrast agent subgroups on both training (0.699 [95%CI: 0.635, 0.764] vs 0.788 [95%CI: 0.707, 0.869]; *p* = 0.101) and validation (0.720 [95%CI: 0.628, 0.813] vs 0.688 [95%CI: 0.569, 0.807]; *p* = 0.709) sets. Calibration curves showed good agreement between the predicted survival by the hybrid model and the observed outcomes on both datasets (Fig. 4B and C).

### Comparisons between the hybrid model and staging systems on the validation set

**Fig. 5:**
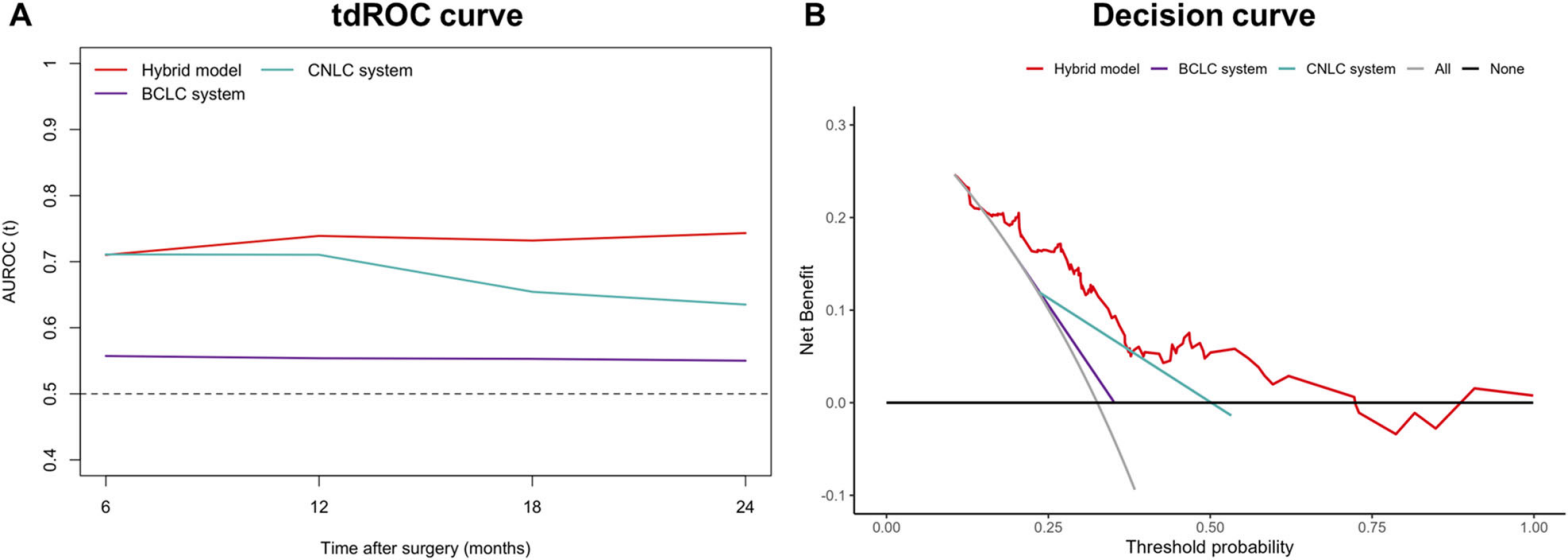
Performance of the hybrid model and staging systems on the validation set. **A** tdROC curves at various time points of the hybrid model and staging systems on the validation set. **B** Decision curves of the hybrid model and staging systems on the validation set. AUROC, areas under the receiver operating characteristic

On the validation set, the C-index of the hybrid model (0.706; 95%CI: 0.630, 0.783) was higher than the BCLC system (0.543; 95%CI: 0.494, 0.592; *p* < 0.001) but showed no evidence of a difference from the CNLC system (0.630; 95%CI: 0.557, 0.703; *p* = 0.061) (Table 4). In addition, the hybrid model (0.710–0.743) demonstrated superior tdAUC to the BCLC system (0.550–0.557; *p* range, 0.005−< 0.001) for predicting early recurrence at 6-, 12-, 18-, and 24-month on the validation set, whereas the model (0.743) yielded a higher tdAUC at 24-month than the CNLC system (0.635; *p* = 0.032) but showed no evidence of a difference at other time points (*p* range, 0.166–0.992) (Table 4; Fig. 5A). Decision curves revealed that the hybrid model provided a larger net benefit than two staging systems on the validation set (Fig. 5B).

**Table 4:** Comparison of performance between the hybrid model and staging systems on the validation Set (*n* = 129)

Performance	Hybrid model	BCLC system	*p* value	CNLC system	*p* value
**C-index**	0.706 (0.630, 0.783)	0.543 (0.494, 0.592)	**< 0.001**	0.630 (0.557, 0.703)	0.061
**6-month tdAUC**	0.710 (0.579, 0.842)	0.557 (0.488, 0.627)	**0.005**	0.711 (0.590, 0.832)	0.992
**12-month tdAUC**	0.739 (0.638, 0.840)	0.554 (0.490, 0.618)	**< 0.001**	0.710 (0.610, 0.811)	0.635
**18-month tdAUC**	0.732 (0.634, 0.830)	0.553 (0.492, 0.614)	**< 0.001**	0.654 (0.561, 0.748)	0.166
**24-month tdAUC**	0.743 (0.653, 0.834)	0.550 (0.489, 0.611)	**< 0.001**	0.635 (0.547, 0.723)	**0.032**

Statistically significant *p* values are bold

Data in parentheses are 95% confidence intervals

### Early recurrence risk stratification on the training and validation sets

**Fig. 6:**
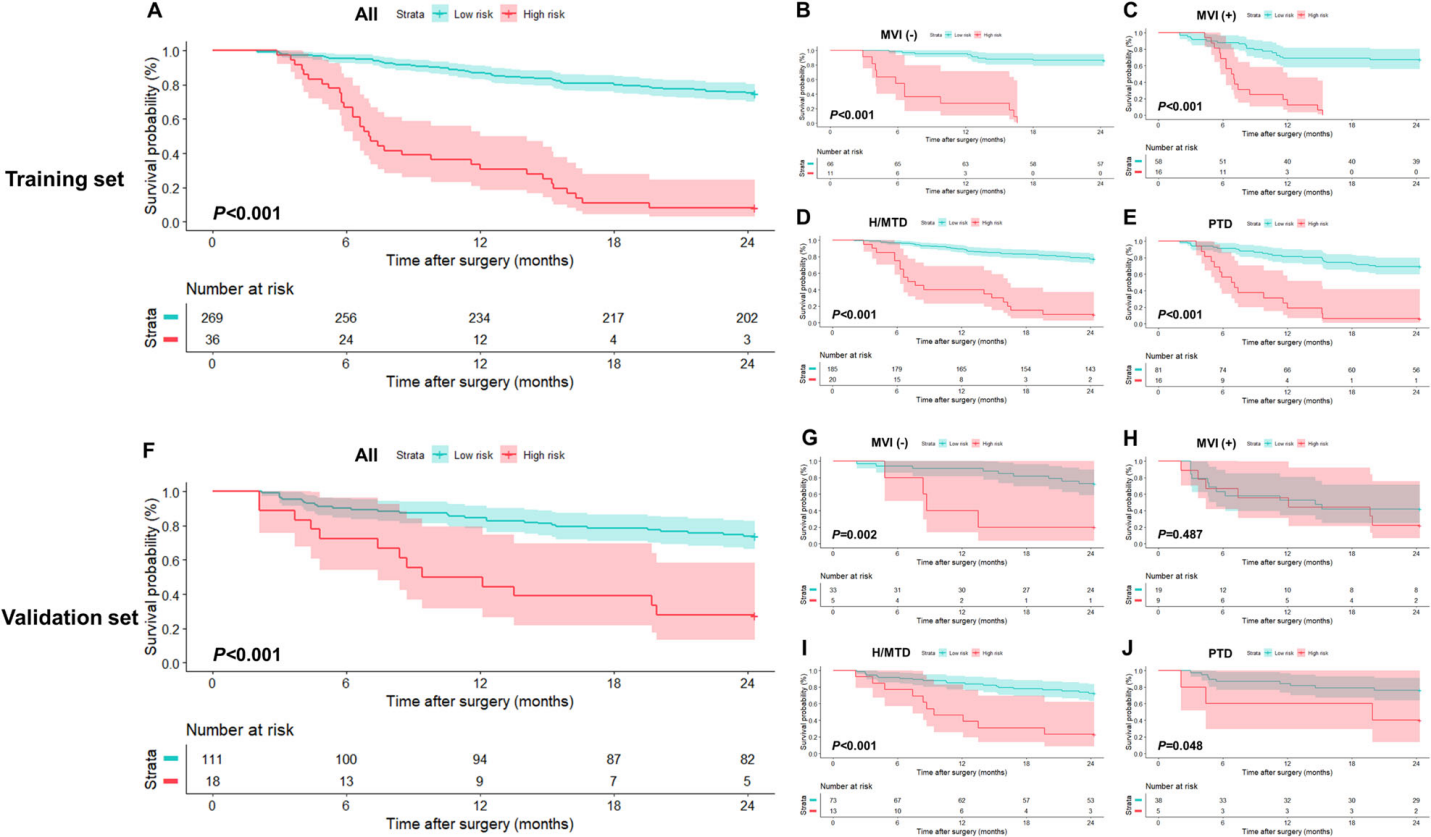
Kaplan-Meier curves demonstrating differences in RFS between low (< 1.25) and high (≥ 1.25) risk strata defined by the hybrid model in all patients on the (**A**) training and (**F**) validation sets, respectively. Similar results were observed in the (**B**) MVI absent, (**C**) MVI present, (**D**) H/MTD, and (**E**) PTD subgroups on training set. Except for the (**H**) MVI present subgroup, two prognostically distinct risk strata were also obtained in (**G**, **I**, **J**) other subgroups on the validation set. H/MTD, high/moderate tumor differentiation; PTD poor tumor differentiation

Using 1.25 as the cutoff score derived by the X-tile analysis, the hybrid model stratified all patients on the training set into two risk strata: low risk (< 1.25; *n* = 269; RFS rate at 24-month, 75.1%), and high risk (≥ 1.25; *n* = 36; RFS rate at 24-month, 8.3%) (*p* < 0.001) (Table 5; Fig. 6A). In each subgroup, including absence and presence of MVI, and high or moderate tumor differentiation and poor tumor differentiation, low-risk patients had significantly longer RFS than high-risk patients on the training set (*p* < 0.001 for all) (Table 5; Fig. 6B–E).

**Table 5:** RFS rates and HRs according to each risk group defined by the hybrid model in all patients and two pathological subgroups

Dataset and risk group	No. of patient	Event	RFS Rate at 12-month, %	RFS rate at 24-month, %	HR	*p* value
**Training set**						
All						**< 0.001**
Low risk	269	67	87.0 (83.1, 91.1)	75.1 (70.1, 80.4)	Reference	
High risk	36	33	30.6 (18.7, 50.0)	8.3 (2.8, 24.6)	8.588 (5.561, 13.263)	
MVI absent						**< 0.001**
Low risk	66	9	95.5 (90.6, 100.0)	86.4 (78.5, 95.1)	Reference	
High risk	11	11	27.3 (10.4, 71.6)	0.0 (NA, NA)	19.797 (7.706, 50.860)	
MVI present						**< 0.001**
Low risk	58	19	69.0 (58.0, 82.0)	67.2 (56.2, 80.5)	Reference	
High risk	16	16	12.5 (3.4, 45.7)	0.0 (NA, NA)	6.105 (3.028, 12.307)	
High or moderate tumor differentiation						**< 0.001**
Low risk	185	42	89.2 (84.8, 93.8)	77.3 (71.5, 83.6)	Reference	
High risk	20	18	40.0 (23.4, 68.4)	10.0 (2.7, 37.2)	8.901 (5.029, 15.754)	
Poor tumor differentiation						**< 0.001**
Low risk	81	25	81.5 (73.4, 90.4)	69.1 (59.8, 80.0)	Reference	
High risk	16	15	18.8 (6.8, 52.0)	6.3 (0.9, 41.7)	7.100 (3.592, 14.037)	
**Validation set**						
All						**< 0.001**
Low risk	111	29	84.7 (78.2, 91.7)	73.9 (66.1, 82.5)	Reference	
High risk	18	13	50.0 (31.5, 79.4)	27.8 (13.2, 58.5)	4.148 (2.143, 8.031)	
MVI absent						**0.002**
Low risk	33	9	90.9 (81.6, 100.0)	72.7 (59.0, 89.6)	Reference	
High risk	5	4	40.0 (13.7, 100.0)	20.0 (3.5, 100.0)	5.836 (1.707, 19.954)	
MVI present						0.487
Low risk	19	11	52.6 (34.4, 80.6)	42.1 (24.9, 71.3)	Reference	
High risk	9	7	55.6 (31.0, 99.7)	22.2 (6.5, 75.4)	1.395 (0.540, 3.602)	
High or moderate tumor differentiation						**< 0.001**
Low risk	73	20	84.9 (77.1, 93.5)	72.6 (63.1, 83.6)	Reference	
High risk	13	10	46.2 (25.7, 83.0)	23.1 (8.6, 62.3)	4.517 (2.086, 9.781)	
Poor tumor differentiation						**0.048**
Low risk	38	9	84.2 (73.4, 96.6)	76.3 (63.9, 91.1)	Reference	
High risk	5	3	60.0 (29.3, 100.0)	40.0 (13.7, 100.0)	3.467 (0.936, 12.846)	

Statistically significant *p* values are bold

Data in parentheses are 95%CIs

*NA* not available

Based on the above threshold, two risk strata with significantly different RFS were also obtained for all patients on the validation set (RFS rate at 24-month: 73.9% vs. 27.8%; *p* < 0.001) (Table 5; Fig. 6F). Additionally, low-risk patients showed significantly longer RFS compared with high-risk patients in subgroups of MVI absence (*p* = 0.002) (Table 5; Fig. 6G), high or moderate tumor differentiation (*p* < 0.001) (Table 5; Fig. 6I), and poor tumor differentiation (*p* = 0.048) (Table 5; Fig. 6J) on the validation set. However, there was no evidence of a difference in RFS between low- and high-risk patients in the MVI present subgroup (*p* = 0.487) on the validation set (Table 5; Fig. 6H).

Representative images of a patient with HCC at high risk of early recurrence determined by the hybrid model are shown in Fig. S2.

## Discussion

In this study, using DL-assisted automated segmentation, we developed and validated a hybrid model, which integrated MRI radiomic signature and two imaging features (rim APHE and incomplete tumor “capsule”), to predict early recurrence for patients with single HCC following curative resection. The model demonstrated superior prognostic accuracy (validation set 2-year tdAUC, 0.743) and better clinical utility compared to the BCLC (0.550; *p* < 0.001) and CNLC (0.635; *p* = 0.032) systems. Moreover, patients in the model-predicted high-risk group exhibited worse RFS than those in the low-risk group.

Accurate and reproducible segmentation is the cornerstone of radiomic analyses. However, most existing radiomics-based assessment of HCC recurrence utilized manual or semiautomated segmentation, reducing both efficiency and reproducibility. In our study, the consistency between the automated and manual tumor segmentations was satisfactory, with the mean DSC of 0.85 ± 0.12. Moreover, in a manual segmentation-based study comprising 167 single HCC, Kim et al reported that a combined clinicopathologic-radiomic model with the 3-mm border extension achieved the highest performance (C-index: 0.716) for predicting early postsurgical recurrence [6]. In our study, the automated segmentation-based hybrid model achieved similar validation set discrimination (C-index: 0.706). These initial findings offered a promising prospect for using DL-assisted automated segmentation, which might be more effective and reproducible, to standardize the development of radiomic models, thereby facilitating their translation into clinical practice.

Recently, a few studies have shown the potential utility of automated segmentation-based CT or MRI radiomics in predicting postsurgical recurrence of HCC [28, 29]. For instance, Wang et al employed a DL model to automatically segment tumors on arterial phase images in the external cohort (*n* = 31) and reported that an MRI-based radiomic-clinical model achieved good accuracy for predicting postsurgical recurrence [29]. To the best of our knowledge, our study is the first study in the literature to use DL-assisted automated segmentations of both the liver and tumor for MRI radiomics-based evaluation of HCC early recurrence. In comparison to previous studies, our research included a larger number of patients (*n* = 434), thereby enhancing the reliability and robustness of the results presented. In addition, to determine the optimal radiomic signature, we comprehensively investigated the prognostic impact of tumor, tumor border extensions, and the liver. Therefore, our final radiomic signature may convey more abundant information to achieve accurate prognostication.

The biological rationales underlying the association between the hybrid model and HCC early recurrence are not fully understood. In our study, the radiomic signature of *HCC with 5* *mm tumor border extension and liver* exhibited the highest performance for predicting early recurrence of HCC. We speculated that this model may comprehensively capture the whole spectrums of aggressive tumor features, peritumoral microenvironments, and liver morphological and functional characteristics. Rim APHE has been shown to be correlated with tumor aggressiveness, e.g., proliferative subtype, MVI, hypoxic and fibrotic tumor microenvironments, and increased stemness of HCC [30–32]. An incomplete tumor “capsule”, indicative of infiltrative tumor growth, has been identified as an imaging marker for predicting MVI and high *BRAF* and *RAF1* expression in HCC, which can promote tumor invasion and metastasis [33–35].

Remarkably, a small subset of our cases (*n* = 40) encountered challenges during automated segmentation and were excluded from radiomic analyses. These inaccurate segmentations could arise from various factors, such as the inhomogeneous signal intensity within the tumor, the obscured tumor margin, and additional signal interferences introduced by peritumoral liver parenchyma, adjacent benign cysts, or organs (e.g., heart, gall bladder, and right kidney). Hence, further refinement of the DL algorithm is warranted to improve the automated segmentation performance. If its accuracy and generalizability can be further validated and improved on large-scale multicenter populations, this advanced technique would probably become an applicable workflow in routine clinical practice.

Our study had several limitations. First, the retrospective design may have led to unavoidable selection bias. Second, the hybrid model was not validated in an independent external cohort, which is crucial to check the generalizability of the model. Thus, future multicenter studies are needed to test the applicability of our model in different populations. Third, the interobserver agreement for incomplete tumor “capsule” was only fair (Cohen’s κ coefficient, 0.316), which may reduce the reproducibility of the model. However, as an intrinsic limitation of subjective manual assessment, future studies are warrant to identify approaches for reducing interreader variability, such as utilizing more standardized image criteria or development of computer-aided feature interpretation. Fourth, the vast majority of included patients (95.4%) had HBV infection; hence, our results may not pertain to patients with other etiologies (e.g., hepatitis C virus infection and alcohol abuse). Fifth, we did not construct a postoperative model for predicting HCC early recurrence due to a large number of missing data for key pathological features (e.g., MVI and tumor differentiation). Nonetheless, the purpose of our study was to establish a noninvasive model for assisting in clinical decision-making before treatment. Finally, we did not investigate the prognostic risk stratification ability of the hybrid model across different pathological subtypes of HCC due to insufficient data. Future radiomic studies are encouraged to look at this issue.

In conclusion, using DL-assisted automated segmentation, we proposed a hybrid model by incorporating MRI-based radiomic signature, rim APHE, and incomplete tumor “capsule” for prediction of HCC early recurrence after resection. The model demonstrated superior predictive performance than two widely used staging systems, holding the potential to facilitate individualized risk estimation of postsurgical early recurrence in a single HCC.

### Supplementary information

Electronic Supplementary Material

## Abbreviations

AI: Artificial intelligence
APHE: Arterial phase hyperenhancement
BCLC: Barcelona clinic liver cancer
C-index: Concordance index
CNLC: Chinese National Liver Cancer
DL: Deep learning
DSCs: Dice similarity coefficients
HCC: Hepatocellular carcinoma
HR: Hazard ratio
IQR: Interquartile range
MRI: Magnetic resonance imaging
MVI: Microvascular invasion
RFS: Recurrence-free survival
RSF: Random survival forest
tdAUC: Time-dependent area under the receiver operating characteristic curve
tdROC: Time-dependent receiver operating characteristic
VIF: Variance inflation factor
VOI: Volume of interest
