# Supplementary material for: MRI radiomics based on deep learning automated segmentation to predict early recurrence of hepatocellular carcinoma
Source: Insights Imaging. 2024 May 20;15:120. doi: 10.1186/s13244-024-01679-8 (PMC11102894; doi:10.1186/s13244-024-01679-8)
Supplement: Supplementary file 1 — Electronic Supplementary Material [file 13244_2024_1679_MOESM1_ESM.pdf]

**MRI radiomics based on deep learning automated segmentation to predict early recurrence of hepatocellular carcinoma**

**ELECTRONIC SUPPLEMENTARY MATERIAL**

**Supplementary Material 1** MRI Technique (Page 2)

**Supplementary Material 2** Deep Learning Algorithms for Automated Segmentation on MRI (Page 3)

**Supplementary Material 3** Detailed Methods of Radiomic Analysis (Page 7)

**Table S1** MRI Sequences and Parameters (Page 12)

**Table S2** Dice Similarity Coefficients between the Automated and Manual Tumor Segmentations in 30 randomly selected HCC lesions (Page 15)

**Table S3** Number of Radiomic Features in Each Step of Feature Selection on the Training Set (Page 16)

**Table S4** Eight Groups of Radiomic Signatures for Prediction of Early Recurrence according to Multivariable Cox Regression Analyses (Page 17)

**Figure S1** 3D-Unet Architectures of Liver and Tumor Segmentation Models (Page 18)

**Figure S2** Representative Images of A Patient with HCC at High Risk of Early Recurrence Determined by the Hybrid Model (Page 19)

**Supplementary Material 1** MRI Technique

Magnetic resonance imaging (MRI) with hepatobiliary contrast agent (HCA) was conducted using four 3.0-T systems (GE SIGNA™ Architect; GE SIGNA™ Premier; GE Discovery MR 750; Siemens MAGNETOM Skyra) and one 1.5-T system (uMR588). Additionally, MRI with extracellular contrast agent (ECA) was performed with five 3.0-T systems (Siemens MAGNETOM Skyra; Siemens TrioTim; GE SIGNA™ Architect; GE Discovery MR 750; Philips Ingenia Elition X) and two 1.5-T systems (Siemens Avanto; uMR588). The liver MRI protocols consisted of T2-weighted imaging, diffusion-weighted imaging (b values: 0-1200 s/mm$^2$) with apparent diffusion coefficient (ADC) maps, T1-weighted in- and opposed-phase imaging, and dynamic T1- weighted imaging before and after injection of contrast agent in the late arterial phase, portal venous phase (60 s), delayed phase (ECA MRI; 180s) or transitional phase (HCA MRI; 180 s), and hepatobiliary phase (HCA MRI; 20 minutes). The arterial phase images were obtained either by the acquisition triggered 7 s after arrival of the contrast bolus in the celiac trunk or a multiple arterial phase (MAP) imaging technique. In specific, the MAP images were obtained with an 18 s breath hold 20 s after the contrast agent injection, and further reconstructed with a temporal resolution of 3 s. For HCA MRI, gadoxetate disodium (Primovist®; Bayer Schering Pharma AG) was administered intravenously at 1.0-2.0 ml/s (0.025 mmol/kg of body weight), followed immediately by a 20-30 ml saline flush. For ECA MRI, gadopentetate dimeglumine (Magnevist®; Bayer Schering Pharma AG) or gadoterate meglumine (Dotarem®; Guerbet) or gadobenate dimeglumine (MultiHance®; Bracco) was intravenously administered at 2.5 ml/s (0.1 mmol/kg of body weight). MRI sequences and parameters are detailed in **Table S1.**

**Supplementary Material 2** Deep Learning Algorithms for Automated Segmentation on MRI

**Dataset for the Development of Automated Segmentation Models**

To develop automated deep learning (DL) segmentation models, a total of 1889 patients with focal liver lesions (i.e., hepatocellular carcinoma, hemangioma, and hepatic cyst) from six tertiary hospital in China between December 2013 and February 2021 were included. Patients were allocated into the training set (n=1511), validation set (n=189), and test set (n=189) at a ratio of 8:1:1. Magnetic resonance (MR) images in DICOM format were exported from the picture archiving and communication system. Two abdominal radiologists, both with 5 years of experience in liver MRI, performed manual segmentation of FLLs on T2-weighted imaging, diffusion-weighted imaging (b value of 800 s/mm$^2$), in and opposed phase imaging, pre- and post-contrast enhanced T1-weighted imaging during late arterial phase, portal venous phase, delayed phase (MRI with extracellular contrast agent), transitional phase and hepatobiliary phase (MRI with hepatobiliary contrast agent), avoiding intrahepatic vasculatures. Each radiologist segmented 944 and 945 patients, respectively. To facilitate quality control for manual segmentation, all regions of interest (ROIs) were inspected by a senior radiologist with 30 years of experience in liver MRI. In cases where segmentations were deemed unqualified, manual adjustments were carried out by two junior radiologists. The resulting sketched images were used as input data for training the automated segmentation models.

**Automated Segmentation Model Training, Validation and Test**

The automated segmentation models were trained using a sequential modular approach. Initially, a three-dimensional convolutional neural network (3D-CNN) model [1] was employed to generate a liver segmentation mask. During this phase, the algorithm defined the liver region on MR images, isolating it from adjacent abdominal organs to enable a focused analysis. This algorithm involved an encoder-decoder architecture with 3D convolutions and pooling layers, complemented by the Rectified Linear Unit (RLU) activation and batch normalization. Skip connections were built between corresponding layers of the encoder and decoder. The output layer included

two branches for liver boundary segmentation and pixel-level liver region segmentation, respectively. Following precise liver segmentation and anatomical delineation, rigorous image registration was implemented by aligning multiple MRI sequences with a standardized spatial reference framework, thereby enhancing spatial coherence between the liver segmentation masks across diverse MRI sequences.

To improve FLL detection accuracy, segmented liver images were transformed into input data for a lesion detection algorithm. Subsequently, an advanced deep learning algorithm was developed for automated FLL detection in each sequence of contrast-enhanced MR images. The core algorithm is the use of a 3D-CNN model known as the Unified Multi-Sequence Lesion Detector (MSLD), which comprised two primary elements: *(a)* a series of Single Lesion Detectors (SLD) for independent lesion detection in each sequence, and *(b)* a False Positive Reduction (FPR) module to mitigate false alarms in identified lesions. Utilizing the MSLD model, each detected lesion was annotated by a bounding box in each sequence.

To address MRI sequence diversity, we devised Single Lesion Detectors (SLDs) tailored for each sequence, extending the Mask region-based convolutional neural network (R-CNN) [2] framework to process 3D input images. Four SLDs, sharing the same architecture, effectively accommodated variations in tissue appearances across various sequence groups, including pre-contrast T1WI, post-contrast T1WI, T2WI, and DWI. The SLD framework incorporated the Region Proposal Network (RPN), ROI alignment, lesion identification, and segmentation modules. The introduction of an adaptive receptive field enabled global feature extraction within slices, and the Feature Pyramid Network (FPN) [3] captured multi-scale information for robust perceptual capabilities. Training involved the normalization of preprocessed images (2×2×2 $mm^3$ spacing), cropping into 160×160×160 patches, and employing the Adam optimizer for 200 epochs with a batch size of eight. The initial learning rate was 0.001, decaying every 50 epochs.

Multiple sequences were utilized to minimize the impact of image artifacts on lesion detection. In automated FLL detection within the SLD section, bounding boxes for various sequences were cross-referenced to identify candidate lesions. To reduce

false alarms from artifacts, a dedicated FPR module integrated a 3D-CNN for feature extraction from each ROI, followed by feature integration from multiple sequences for binary predictions. Standardizing ROI dimensions to 32×32×32 ensured uniformity for typical lesion sizes. Model training spanned 200 epochs with an initial learning rate of 0.001, decayed by 0.1 every 30 epochs, and a batch size of 64 for optimized training.

Lesion segmentation was achieved using a 3D-UNet framework, characterized by an encoder-decoder architecture with 3D convolutions and pooling layers. This framework was incorporated Rectified Linear Unit (RLU) activation and batch normalization. The encoder and decoder, each comprising four layers of 3Dconv-bn-RLU, were interconnected. After the final decoder layer, a 3D conv-bn-RLU layer was integrated for the ultimate lesion segmentation prediction. The model employed the Adam optimizer with an initial learning rate of 0.001, gradually reduced by a factor of 0.1 every 30 epochs, culminating after 60 training epochs.

Validation set was utilized to fine-tune the hyperparameters, such as adjusting the learning rate and batch size. This iterative process enabled the identification of optimal hyperparameters that led to best results on a new data set, which played a crucial role in enhancing the model's generalizability.

The accuracy of the automated liver and lesion segmentation models was evaluated on the test set. The mean Dice similarity coefficient (DSC) between the automated and manual liver segmentations was 0.95±0.11, with a range of 0.79 to 0.99 across all sequences. In addition, the mean DSC between the automated and manual lesion segmentations was 0.78±0.16, with a range of 0.59 to 0.96 across all sequences.

## References


1. Han X, Wu X, Wang S, et al (2022) Automated segmentation of liver segment on portal venous phase MR images using a 3D convolutional neural network. Insights Imaging 13:26.
2. He K, Gkioxari G, Dollár P, Girshick R (2017) Mask R-CNN. 2017 IEEE International Conference on Computer Vision (ICCV). p. 2980-2988.
3. Lin TY, Doll ́ar P, Girshick R, He K, Hariharan B, and Belongie S (2017) Feature

Pyramid Networks for Object Detection. 2017 IEEE Conference on Computer Vision and Pattern Recognition (CVPR). p. 936-944.

**Supplementary Material 3** Detailed Methods of Radiomic Analysis

***Image Acquisition, Preprocessing and Automated Segmentation***

De-identified magnetic resonance (MR) images were uploaded to a commercial visualization and analysis software (LiverMRDoc; version 2.10.0; Shukun Technology Co., Ltd).

Before automated segmentation, one radiologist (HW) inspected all MR images in terms of the sequence names, HCC lesions, and corresponding 3D bounding boxes (i.e., the automated lesion detection annotation) on the AI software platform. To ensure accurate localization of tumors, manual adjustment was conducted for 16 patients with inaccurate 3D bounding boxes (e.g., failing to detect HCC lesions or delineate the whole tumors).

Using 3D U-net-based DL algorithms as detailed in **Supplementary Material 2 and Fig. S1**, automated segmentation of liver and HCC lesions was conducted on each transverse section of T2-weighted imaging (T2WI), in phase (IP), opposed phase (OP), arterial phase (AP), portal venous phase (PVP), and delayed phase (DP; for MRI with extracellular contrast agent [ECA]) or translational phase (TP; for MRI with hepatobiliary contrast agent [HCA]) images.

To implement the quality control, one radiologist (HW) visually inspected each segmented tumor and liver, and those (n=40) with inaccurate tumor or liver segmentations on any above sequences were excluded from radiomic analyses. The exclusion criteria for inaccurate segmentation were *(a)* tumor region of interest (ROI) covered nontumoral areas (e.g., liver parenchyma, benign cysts, adjacent organs or tissues) (n=18); *(b)* tumor ROI failed to cover the whole tumor areas (n=8); *(c)* liver ROI failed to cover the whole tumor or liver areas (n=6); and *(d)* liver ROI covered areas beyond the liver (n=8). Examples of inaccurate image segmentations are presented in **Fig. 2.** Manual adjustment was not considered because the study aimed to examine the prognostic utility of this **automated** technique.

To assess the accuracy of automated DL segmentation, one radiologist (TYZ) who was unknown to the automated segmentation results manually segmented 30 randomly chosen HCC lesions using ITK-SNAP (version 3.8.0; www.itksnap.org).

To extract radiomic features of peritumoral areas, the tumor's 3D mask was expanded radially outwards by 5 mm, 10 mm, and 20 mm on each sequence using a medical research platform (UltraScholar, Version 2.0, Shukun Technology Co., Ltd, https://medresearch.shukun.net/). Accordingly, five types of volumes of interest (VOIs) were created: *(a)* tumor VOI, defined as the VOI covering HCC lesion; *(b)* three extended tumor VOIs, defined as the tumor VOI with automated extension of tumor boundaries by 5 mm, 10 mm, and 20 mm, respectively; and *(c)* liver VOI, defined as the VOI covering nontumoral liver parenchyma (**Fig. 3**).

***Radiomic Feature Extraction***

MR image preprocessing and radiomic feature extraction were performed with the PyRadiomic package (version 3.0.1; https://pyradiomic.readthedocs.io/en/v3.0.1/). Before extracting radiomic features, voxels in each MR image volume were resampled to an isotropic voxel size of 1.0 × 1.0 × 1.0 $mm^3$. This standardization helped minimize the impact of various MR imaging conditions, like pixel spacings and slice thicknesses. For gray value discretization, a bin width of 25 was applied to volume images. A normalization of image intensity values was performed to enhance comparability and interpretability of the radiomic features. The normalization scale parameter was set to 1 to retain the original scale of normalized images and ensure the data integrity. To address geometric differences in liver MRI images, a geometry tolerance parameter of 1e-5 was employed during extracting radiomic features.

Extracted feature classes included the shape features, first-order features, second-order features, and higher-order features. The shape-based features described the area, volume, perimeter, contours irregularity and compactness ($perimeter^2$/area) of tumor or liver. The first-order statistics depicted the distribution of individual voxel-values within the MR image without emphasizing their spatial relationships. The second-order features (i.e., Gray Level Co-occurrence Matrix [GLCM] features) conveyed additional information about texture by considering relationships between intensities of neighboring voxel pairs. The higher-order features (e.g., Gray Level Run Length Matrix [GLRLM] features, Gray Level Dependence Matrix [GLDM] features, Neighboring Gray Tone Difference Matrix [NGTDM] features, Gray Level Size Zone

Matrix [GLSZM] features, and Gray Level Distance Zone Matrix [GLDZM] features) provided sophisticated patterns and textural information, highlighting the relationships among multiple voxels [1].

A total of 1688 features were extracted from each VOI in one sequence: 324 first-order statistics, 14 shape features, 432 second-order features (i.e., GLCM features), and 918 higher-order features (i.e., 252 GLDM features, 288 GLRLM features, 288 GLSZM features, 90 NGTDM features). This led to 10,128 features for each VOI on all six sequences. Radiomic features were extracted for VOI of tumor, tumor border extensions (5 mm, 10 mm, and 20 mm), and liver, respectively.

***Radiomic Feature Normalization and Abnormal Feature Exclusion***

Radiomic feature normalization, abnormal feature exclusion, feature selection, and radiomic signature construction were performed with R software (version 4.3.1; The R Foundation for Statistical Computing).

Values of extracted radiomic features on the training set were normalized with z scores; the means and standard deviations derived from the training set were applied to the feature normalization of the test set. Abnormal features with a variance of 0 were excluded from further analyses. Variance measured the degree of dispersion of values; a variance of 0 suggested that feature values were same in all patients. The number of abnormal features ranged from 1552 to 3142 for all radiomic signatures.

***Feature Selection***

After normalization and excluding abnormal features, we followed a four-step procedure to reduce dimensions and select robust radiomic features on the training set.

First, intervariable collinearity was estimated by Spearman correlation analysis. For radiomic features with a Spearman's rank correlation coefficient >0.8, hierarchical feature clustering was performed to remove redundancy. These features were clustered into 1 to N (defined as the number of features divided by 3) classes, respectively. To determine the optimal number of clusters, the mean Silhouette Coefficient (mSC) was used whereby a higher value denoted a better quality of clustering. The resulting cluster was represented by one feature with largest range of

values among the clustered features. As such, representative features in all clusters plus features with a Spearman's rank correlation coefficient ≤0.8 were entered into further analyses. The cluster configurations and representative features generated from the training set were applied to the test set, because all parameters of the radiomic signatures must be remembered with the model building and applied to the test set with the same threshold.

Subsequently, univariable Cox regression analysis was performed to identify significant radiomic features associated with early recurrence. Features with $P<0.01$ were kept for further analyses.

Next, random survival forest (RSF) was applied to select the top 20 features. The measure of variable importance (VIMP) was used to rank the importance of variables [2], with a higher value indicating a greater importance.

Finally, based on top 20 features derived from the RSF, radiomic signatures were constructed by the multivariable Cox regression analysis using backward elimination approach with five-fold cross-validation.

***Radiomic Signature Development and Validation***

Eight groups of radiomic signatures were built for predicting early recurrence based on different combinations of radiomic features extracted from tumor, tumor border extensions (5mm, 10mm, and 20mm), and liver parenchyma, including: *(a)* HCC, *(b)* HCC with 5 mm tumor border extension, *(c)* HCC with 10 mm tumor border extension, *(d)* HCC with 20 mm tumor border extension, *(e)* HCC and liver, *(f)* HCC with 5 mm tumor border extension and liver, *(g)* HCC with 10 mm tumor border extension and liver, and *(h)* HCC with 20 mm tumor border extension and liver. The optimal radiomic signature that exhibited highest performance was selected for building the hybrid model.

**References**

1. Abbasian Ardakani A, Bureau NJ, Ciaccio EJ, Acharya UR (2022) Interpretation of radiomics features-A pictorial review. *Comput Methods Programs Biomed* 215:106609.

2. Chen X, Ishwaran H (2012) Random forests for genomic data analysis. *Genomics* 99:323-329.

**Table S1** MRI Sequences and Parameters

| Sequence | T1-weighted IP and OP imaging | Dynamic T1-weighted 3D GRE | T2-weighted 2D FSE | Diffusion-weighted imaging† |
|---|---|---|---|---|
| **GE Discovery MR 750 3.0 Tesla (16-channel phased-array torsor coil)** | | | | |
| Repetition time (ms) | 150 | 4.1 | 6315 | 9230 |
| Echo time (ms) | 2.5/1.3 | 1.9 | 78 | Minimum |
| Flip angle (°) | 70 | 15 | 111 | 90 |
| Section thickness (mm) | 6 | 2 | 6 | 6 |
| Spacing (mm) | 2 | - | 2 | 2 |
| Matrix size | 288×192 | 512×512 | 288×244 | 128×128 |
| Field of view ($mm^2$) | 420×420 | 380×300 | 360×280 | 360×380 |
| Acquisition time (s) | 31 | 15 | RG | RG |
| Fat suppression | No | Yes | Yes | Yes |
| **GE SIGNA™ Architect 3.0 Tesla (30-channel body anterior coil)** | | | | |
| Repetition time (ms) | 233.8 | 3.9 | 2400 | 5000 |
| Echo time (ms) | 2.3/1.1 | 1.7 | 85 | Minimum |
| Flip angle (°) | 55 | 15 | 111 | 90 |
| Section thickness (mm) | 7 | 3 | 7 | 7 |
| Spacing (mm) | 2 | - | 2 | 2 |
| Matrix size | 160×288 | 320×240 | 320×192 | 160×128 |
| Field of view ($mm^2$) | 380×323 | 380×380 | 380×304 | 380×342 |
| Acquisition time (s) | 18 | 15 | 34 | RG |
| Fat suppression | No | Yes | Yes | Yes |
| **GE SIGNA™ Premier 3.0 Tesla (30-channel body anterior coil)** | | | | |
| Repetition time (ms) | 146.8 | 3.2 | 2200 | 5000 |
| Echo time (ms) | 2.3/1.1 | 1.4 | 85 | Minimum |
| Flip angle (°) | 55 | 15 | 111 | 90 |
| Section thickness (mm) | 7 | 2.4 | 7 | 7 |
| Spacing (mm) | 2 | - | 2 | 2 |
| Matrix size | 320×192 | 320×240 | 320×224 | 120×240 |
| Field of view ($mm^2$) | 342×380 | 380×380 | 304×380 | 380×380 |
| Acquisition time (s) | 16 | 15 | 47 | RG |
| Fat suppression | No | Yes | Yes | Yes |
| **Siemens MAGNETOM Skyra 3.0 Tesla (18-channel body array coil)** | | | | |
| Repetition time (ms) | 81 | 3.95 | 2160 | 5600 |
| Echo time (ms) | 2.72/1.4 | 1.92 | 100 | 68 |
| Flip angle (°) | 70 | 9 | 160 | 90 |
| Section thickness (mm) | 6 | 2.5 | 6 | 6 |
| Spacing (mm) | 1.8 | - | 1.8 | 1.8 |
| Matrix size | 352×286 | 352×256 | 320×288 | 100×76 |
| Field of view ($mm^2$) | 400×325 | 400×296 | 433×433 | 380×289 |
| Acquisition time (s) | 24 | 14 | 36 | 233 |
| Fat suppression | No | Yes | Yes | Yes |

| | | | | |
|---|---|---|---|---|
| **Siemens TrioTim 3.0 Tesla (8-channel body anterior coil)** | | | | |
| Repetition time (ms) | 181 | 3.47 | 2700 | 5900 |
| Echo time (ms) | 2.2/3.67 | 1.25 | 95 | 76 |
| Flip angle (°) | 65 | 9 | 140 | 90 |
| Section thickness (mm) | 6 | 2.4 | 6 | 6 |
| Spacing (mm) | 7.8 | - | 7.8 | 7.8 |
| Matrix size | 256×131 | 320×133 | 320×147 | 192×154 |
| Field of view ($mm^2$) | 410×269 | 434×257 | 442×254 | 393×393 |
| Acquisition time (s) | 18 | 17 | RG | 245 |
| Fat suppression | No | Yes | Yes | Yes |
| **Siemens Avanto 1.5 Tesla (30-channel body anterior coil)** | | | | |
| Repetition time (ms) | 72 | 5.41 | 2530 | 3600 |
| Echo time (ms) | 4.92/2.22 | 2.39 | 84 | 88 |
| Flip angle (°) | 70 | 10 | 150 | 90 |
| Section thickness (mm) | 6 | 2.5 | 6 | 6 |
| Spacing (mm) | 7.8 | - | 7.8 | 7.8 |
| Matrix size | 256×158 | 320×138 | 256×187 | 192×115 |
| Field of view ($mm^2$) | 328×225 | 382×238 | 293×251 | 310×232 |
| Acquisition time (s) | 16 | 15 | 47 | 92 |
| Fat suppression | No | Yes | Yes | Yes |
| **Siemens Avanto 1.5 Tesla (8-channel body anterior coil)** | | | | |
| Repetition time (ms) | 87 | 5.4 | 2710 | 2000 |
| Echo time (ms) | 4.92/2.22 | 2.38 | 84 | 72 |
| Flip angle (°) | 70 | 10 | 150 | 90 |
| Section thickness (mm) | 7.5 | 2 | 7.5 | 7.5 |
| Spacing (mm) | 9.75 | - | 9.75 | 9.75 |
| Matrix size | 256×187 | 320×131 | 256×177 | 192×125 |
| Field of view ($mm^2$) | 308×380 | 241×407 | 308×380 | 308×379 |
| Acquisition time (s) | 33 | 15 | 27 | 20 |
| Fat suppression | No | Yes | Yes | Yes |
| **Philips Ingenia Elition X 3.0 Tesla (16-channel body anterior coil)** | | | | |
| Repetition time (ms) | 164.53 | 4.20 | 1883.51 | 1653.65 |
| Echo time (ms) | 2.30/1.15 | 0.00 | 90 | 60.29 |
| Flip angle (°) | 50 | 10 | 90 | 90 |
| Section thickness (mm) | 6 | 3 | 6.8 | 7 |
| Spacing (mm) | 7.5 | 1.5 | 8.5 | 8.5 |
| Matrix size | 256×201 | 344×252 | 272×78 | 142×140 |
| Field of view ($mm^2$) | 360×360 | 380×380 | 380×380 | 380×380 |
| Acquisition time (s) | 11 | 13 | 46 | 52 |
| Fat suppression | No | Yes | Yes | Yes |
| **uMR588 1.5 Tesla (6-channel body anterior coil)** | | | | |
| Repetition time (ms) | 117.6 | 4.2 | 2600 | 3350 |
| Echo time (ms) | 4.7/2.27 | 1.88 | 99.2 | 77 |
| Flip angle (°) | 60 | 10 | 90 | 90 |

| | | | | |
|---|---|---|---|---|
| Section thickness (mm) | 6.5 | 2.5 | 6.5 | 6.5 |
| Spacing (mm) | 1.3 | - | 1.5 | 10 |
| Matrix size | 256×174 | 256×154 | 256×168 | 128×92 |
| Field of view (mm$^2$) | 320×400 | 255×400 | 427×320 | 320×400 |
| Acquisition time (s) | 29 | 13 | 39 | RG |
| Fat suppression | No | Yes | Yes | Yes |

FSE, fast spin-echo; GRE, gradient recall echo; IP, in-phase; MRI, magnetic resonance imaging; NA, not available; OP, opposed-phase; RG, respiratory gating; 3D, three-dimensional; 2D, two-dimensional.

†Images were acquired under free breath.

**Table S2** Dice Similarity Coefficients between the Automated and Manual Tumor Segmentations in 30 randomly selected HCC lesions

| Sequence | Dice similarity coefficient | | |
|---|---|---|---|
| | Mean ± SD | Median (IQR) | Range |
| T2WI | 0.89 ± 0.07 | 0.91 (0.89-0.94) | 0.69-0.96 |
| IP | 0.86 ± 0.08 | 0.89 (0.83-0.92) | 0.71-0.94 |
| OP | 0.76 ± 0.21 | 0.84 (0.67-0.91) | 0.16-0.94 |
| AP | 0.87 ± 0.07 | 0.89 (0.86-0.92) | 0.71-0.94 |
| PVP | 0.84 ± 0.10 | 0.87 (0.82-0.89) | 0.46-0.94 |
| DP (ECA MRI) | 0.84 ± 0.08 | 0.86 (0.82-0.91) | 0.63-0.92 |
| TP (HCA MRI) | 0.83 ± 0.24 | 0.93 (0.83-0.94) | 0.17-0.94 |
| All | 0.84 ± 0.13 | 0.88 (0.82-0.92) | 0.16-0.96 |

AP, arterial phase; DP, delayed phase; ECA, extracellular contrast agent; HCA, hepatobiliary contrast agent; HCC, hepatocellular carcinoma; IP, in phase; IQR, interquartile range; MRI, magnetic resonance imaging; OP, opposed phase; PVP, portal venous phase; SD, standard deviation; TP, transitional phase; T2WI, T2-weighted imaging.

**Table S3** Number of Radiomic Features in Each Step of Feature Selection on the Training Set

| Dataset and Group | No. of Original Radiomic Features | No. of Excluded Abnormal Radiomic Features | No. of Radiomic Features Entered into Hierarchical Feature Clustering | No. of Radiomic Features Excluded by Hierarchical Feature Clustering | No. of Radiomic Features Entered into Univariable Cox Regression Analysis | No. of Radiomic Features Excluded by Univariable Cox Regression Analysis | No. of Radiomic Features Entered into Random Survival Forest |
|---|---|---|---|---|---|---|---|
| **Training Set (n=305)** | | | | | | | |
| HCC | 10128 | 1552 | 3946 | 2644 | 5932 | 4187 | 1745 |
| HCC with 5 mm tumor border extension | 10128 | 1553 | 3858 | 2764 | 5811 | 4271 | 1540 |
| HCC with 10 mm tumor border extension | 10128 | 1562 | 3820 | 2750 | 5816 | 4395 | 1421 |
| HCC with 20 mm tumor border extension | 10128 | 1573 | 3596 | 2692 | 5863 | 4583 | 1280 |
| HCC + liver | 20256 | 3121 | 7657 | 5105 | 12030 | 9726 | 2304 |
| HCC with 5 mm tumor border extension + liver | 20256 | 3122 | 7569 | 5342 | 11792 | 9707 | 2085 |
| HCC with 10 mm tumor border extension + liver | 20256 | 3131 | 7531 | 5429 | 11696 | 9747 | 1949 |
| HCC with 20 mm tumor border extension + liver | 20256 | 3142 | 7307 | 4881 | 12233 | 10347 | 1886 |

HCC, hepatocellular carcinoma.

**Table S4** Eight Groups of Radiomic Signatures for Prediction of Early Recurrence according to Multivariable Cox Regression Analyses

| Group | Training Set (n=305) | | | |
|---|---|---|---|---|
| | Top 20 Features Entered into Multivariable Cox Regression Analysis | Radiomic Signature | | |
| | | Feature | Hazard Ratio | *P* Value |
| HCC | [1] T2WI_HCC_logarithm_glszm_Large Area Low Gray Level Emphasis<br>[2] T2WI_HCC_logarithm_glrlm_Long Run Low Gray Level Emphasis<br>[3] T2WI_HCC_squareroot_glrlm_Long Run Low Gray Level Emphasis<br>[4] T2WI_HCC_original_glrlm_Long Run Low Gray Level Emphasis<br>[5] IP_HCC_lbp.2D_glszm_Zone Variance<br>[6] OP_HCC_ logarithm_glszm_Large Area Low Gray Level Emphasis<br>[7] AP_HCC_lbp.3D.k_glcm_Cluster Shade<br>[8] T2WI_HCC_logarithm_glszm_Large Area Emphasis<br>[9] AP_HCC_wavelet.HHL_glszm_Zone Entropy<br>[10] IP_HCC_lbp.3D.m2_glszm_Zone Variance<br>[11] IP_HCC_lbp.3D.m1_glszm_Size Zone Nonuniformity Normalized<br>[12] OP_HCC_lbp.3D.k_glszm_Zone Variance<br>[13] IP_HCC_exponential_glszm_Zone Entropy<br>[14] OP_HCC_square_glszm_Zone Entropy<br>[15] IP_HCC_lbp.2D_glszm_Size Zone Nonuniformity Normalized<br>[16] TP/DP_HCC_wavelet.HLL_glszm_Small Area Low Gray Level Emphasis<br>[17] T2WI_HCC_original_first order_Energy<br>[18] AP_HCC_lbp.3D.k_first order_Range<br>[19] OP_HCC_squareroot_glrlm_Long Run Low Gray Level Emphasis<br>[20] AP_HCC_lbp.3D.k_ngtdm_Contrast | [1] T2WI_HCC_logarithm_glrlm_Long Run Low Gray Level Emphasis<br>[2] IP_HCC_lbp.2D_glszm_Zone Variance<br>[3] AP_HCC_lbp.3D.k_ngtdm_Contrast | 1.504 (1.320, 1.712)<br>1.348 (1.175, 1.547)<br>1.400 (1.223, 1.603) | <0.001<br><0.001<br><0.001 |
| HCC with 5 mm tumor border extension | [1] OP_HCC.peri5mm_lbp.3D.m1_glszm_Gray Level Nonuniformity<br>[2] OP_HCC.peri5mm_square_glszm_Gray Level Nonuniformity<br>[3] T2WI_HCC.peri5mm_wavelet.LLL_glszm_Large Area Low Gray Level Emphasis<br>[4] PVP_HCC.peri5mm_wavelet.HLL_glrlm_Long Run High Gray Level Emphasis<br>[5] IP_HCC.peri5mm_lbp.3D.m1_glszm_Size Zone Nonuniformity Normalized<br>[6] PVP_HCC.peri5mm_wavelet.HHH_glszm_Zone Entropy<br>[7] OP_HCC.peri5mm_lbp.2D_glszm_Gray Level Nonuniformity<br>[8] OP_HCC.peri5mm_square_glszm_Zone Entropy<br>[9] TP/DP_HCC.peri5mm_logarithm_glszm_Zone Percentage<br>[10] OP_HCC.peri5mm_wavelet.HHH_glszm_Size Zone Nonuniformity Normalized<br>[11] AP_HCC.peri5mm_lbp.3D.k_ngtdm_Contrast<br>[12] OP_HCC.peri5mm_lbp.3D.m2_glszm_Zone Entropy<br>[13] OP_HCC.peri5mm_gradient_glszm_Gray Level Nonuniformity<br>[14] OP_HCC.peri5mm_gradient_glszm_Zone Entropy<br>[15] OP_HCC.peri5mm_lbp.3D.m2_glszm_Size Zone Nonuniformity Normalized<br>[16] OP_HCC.peri5mm_lbp.2D_glszm_Zone Entropy<br>[17] IP_HCC.peri5mm_exponential_glszm_Zone Entropy<br>[18] IP_HCC.peri5mm_wavelet.HHL_glszm_Size Zone Nonuniformity Normalized<br>[19] OP_HCC.peri5mm_square_glszm_Size Zone Nonuniformity<br>[20] IP_HCC.peri5mm_wavelet.HHL_glszm_Gray Level Nonuniformity | [1] OP_HCC.peri5mm_lbp.3D.m1_glszm_Gray Level Nonuniformity<br>[2] T2WI_HCC.peri5mm_wavelet.LLL_glszm_Large Area Low Gray Level Emphasis<br>[3] PVP_HCC.peri5mm_wavelet.HLL_glrlm_Long Run High Gray Level Emphasis<br>[4] IP_HCC.peri5mm_lbp.3D.m1_glszm_Size Zone Nonuniformity Normalized<br>[5] OP_HCC.peri5mm_square_glszm_Zone Entropy<br>[6] TP/DP_HCC.peri5mm_logarithm_glszm_Zone Percentage<br>[7] AP_HCC.peri5mm_lbp.3D.k_ngtdm_Contrast<br>[8] OP_HCC.peri5mm_square_glszm_Size Zone Nonuniformity | 0.479 (0.225, 1.019)<br>1.170 (1.045, 1.309)<br>1.582 (1.287, 1.944)<br>0.768 (0.653, 0.903)<br>2.146 (1.036, 4.442)<br>0.476 (0.192, 1.180)<br>1.495 (1.236, 1.808)<br>1.328 (1.116, 1.580) | 0.056<br>0.006<br><0.001<br>0.001<br>0.040<br>0.109<br><0.001<br>0.001 |
| HCC with 10 mm tumor border extension | [1] OP_HCC.peri10mm_wavelet.HHL_glszm_Zone Entropy<br>[2] AP_HCC.peri10mm_wavelet.LHL_glszm_Zone Entropy<br>[3] IP_HCC.peri10mm_square_glszm_Zone Entropy<br>[4] OP_HCC.peri10mm_wavelet.HHL_glszm_Gray Level Nonuniformity<br>[5] IP_HCC.peri10mm_wavelet.HHL_glszm_Size Zone Nonuniformity Normalized<br>[6] OP_HCC.peri10mm_square_glszm_Zone Variance<br>[7] OP_HCC.peri10mm_lbp.2D_glszm_Zone Variance<br>[8] IP_HCC.peri10mm_exponential_glszm_Size Zone Nonuniformity Normalized<br>[9] OP_HCC.peri10mm_wavelet.HHL_glszm_Size Zone Nonuniformity Normalized<br>[10] IP_HCC.peri10mm_lbp.3D.m1_glszm_Size Zone Nonuniformity Normalized<br>[11] AP_HCC.peri10mm_wavelet.LHH_glcm_Sum Entropy<br>[12] TP/DP_HCC.peri10mm_wavelet.LHH_glcm_Sum Entropy<br>[13] OP_HCC.peri10mm_lbp.3D.m1_glszm_Zone Variance<br>[14] IP_HCC.peri10mm_lbp.3D.m1_glszm_Zone Entropy<br>[15] OP_HCC.peri10mm_lbp.3D.m2_glszm_Zone Variance<br>[16] PVP_HCC.peri10mm_wavelet.HLL_first order_Median<br>[17] OP_HCC.peri10mm_wavelet.LLH_glszm_Zone Entropy<br>[18] T2WI_HCC.peri10mm_wavelet.HHH_glszm_Small Area Low Gray Level Emphasis<br>[19] IP_HCC.peri10mm_wavelet.LHH_glszm_Size Zone Nonuniformity<br>[20] AP_HCC.peri10mm_exponential_first order_Kurtosis | [1] OP_HCC.peri10mm_wavelet.HHL_glszm_Zone Entropy<br>[2] AP_HCC.peri10mm_wavelet.LHL_glszm_Zone Entropy<br>[3] OP_HCC.peri10mm_square_glszm_Zone Variance<br>[4] AP_HCC.peri10mm_wavelet.LHH_glcm_Sum Entropy<br>[5] TP/DP_HCC.peri10mm_wavelet.LHH_glcm_Sum Entropy<br>[6] PVP_HCC.peri10mm_wavelet.HLL_first order_Median<br>[7] OP_HCC.peri10mm_wavelet.LLH_glszm_Zone Entropy<br>[8] T2WI_HCC.peri10mm_wavelet.HHH_glszm_Small Area Low Gray Level Emphasis<br>[9] IP_HCC.peri10mm_wavelet.LHH_glszm_Size Zone Nonuniformity<br>[10] AP_HCC.peri10mm_exponential_first order_Kurtosis | 1.347 (1.017, 1.783)<br>1.296 (1.005, 1.670)<br>1.188 (1.041, 1.355)<br>0.766 (0.622, 0.944)<br>0.793 (0.636, 0.988)<br>2.235 (1.500, 3.328)<br>1.350 (1.001, 1.821)<br>1.246 (1.006, 1.542)<br>0.849 (0.682, 1.056)<br>1.213 (1.065, 1.382) | 0.038<br>0.045<br>0.010<br>0.012<br>0.039<br><0.001<br>0.049<br>0.043<br>0.141<br>0.004 |
| HCC with 20 mm tumor border extension | [1] T2WI_HCC.peri20mm_wavelet.HHL_glszm_Size Zone Nonuniformity<br>[2] PVP_HCC.peri20mm_wavelet.HHH_gldm_Dependence Entropy<br>[3] PVP_HCC.peri20mm_wavelet.LHH_glcm_Autocorrelation<br>[4] PVP_HCC.peri20mm_wavelet.HHL_first order_Minimum<br>[5] T2WI_HCC.peri20mm_wavelet.LHL_glrlm_Short Run Emphasis<br>[6] PVP_HCC.peri20mm_lbp.3D.k_glrlm_Low Gray Level Run Emphasis<br>[7] PVP_HCC.peri20mm_lbp.3D.k_glrlm_Run Length Nonuniformity<br>[8] AP_HCC.peri20mm_wavelet.HHL_glrlm_Long Run High Gray Level Emphasis<br>[9] PVP_HCC.peri20mm_wavelet.HLL_gldm_Gray Level Variance<br>[10] T2WI_HCC.peri20mm_lbp.2D_first order_90 Percentile<br>[11] PVP_HCC.peri20mm_wavelet.HLL_first order_Entropy<br>[12] AP_HCC.peri20mm_wavelet.LHH_glcm_Sum Entropy<br>[13] PVP_HCC.peri20mm_wavelet.LHH_gldm_Dependence Variance<br>[14] T2WI_HCC.peri20mm_wavelet.HLL_glrlm_Run Variance<br>[15] PVP_HCC.peri20mm_wavelet.HHH_gldm_Dependence Nonuniformity Normalized<br>[16] T2WI_HCC.peri20mm_wavelet.LHH_glrlm_Short Run Low Gray Level Emphasis<br>[17] AP_HCC.peri20mm_lbp.3D.k_ngtdm_Strength<br>[18] T2WI_HCC.peri20mm_wavelet.LHH_glrlm_Run Entropy<br>[19] OP_HCC.peri20mm_wavelet.LLH_glszm_Size Zone Nonuniformity Normalized<br>[20] AP_HCC.peri20mm_wavelet.LHH_glcm_Autocorrelation | [1] T2WI_HCC.peri20mm_wavelet.HHL_glszm_Size Zone Nonuniformity<br>[2] PVP_HCC.peri20mm_wavelet.HHH_gldm_Dependence Entropy<br>[3] PVP_HCC.peri20mm_wavelet.LHH_glcm_Autocorrelation<br>[4] PVP_HCC.peri20mm_wavelet.HHL_first order_Minimum<br>[5] PVP_HCC.peri20mm_lbp.3D.k_glrlm_Run Length Nonuniformity<br>[6] AP_HCC.peri20mm_wavelet.HHL_glrlm_Long Run High Gray Level Emphasis<br>[7] PVP_HCC.peri20mm_wavelet.HLL_gldm_Gray Level Variance<br>[8] T2WI_HCC.peri20mm_lbp.2D_first order_90 Percentile<br>[9] AP_HCC.peri20mm_wavelet.LHH_glcm_Sum Entropy<br>[10] PVP_HCC.peri20mm_wavelet.HHH_gldm_Dependence Nonuniformity Normalized<br>[11] OP_HCC.peri20mm_wavelet.LLH_glszm_Size Zone Nonuniformity Normalized | 1.328 (1.059, 1.666)<br>49.810 (3.959, 626.597)<br>0.706 (0.535, 0.932)<br>0.771 (0.588, 1.012)<br>0.776 (0.594, 1.014)<br>1.392 (1.108, 1.748)<br>3.086 (1.229, 7.748)<br>0.705 (0.572, 0.870)<br>0.746 (0.586, 0.949)<br>43.592 (3.528, 538.630)<br>0.738 (0.548, 0.992) | 0.014<br>0.002<br>0.014<br>0.061<br>0.063<br>0.004<br>0.016<br>0.001<br>0.017<br>0.003<br>0.044 |
| HCC + liver | [1] T2WI_HCC_original_glrlm_Long Run Low Gray Level Emphasis<br>[2] T2WI_HCC_logarithm_glrlm_Long Run Low Gray Level Emphasis<br>[3] T2WI_HCC_squareroot_glrlm_Long Run Low Gray Level Emphasis<br>[4] PVP_Liver_wavelet.LHH_glrlm_Run Length Nonuniformity Normalized<br>[5] PVP_Liver_wavelet.LHH_glcm_Sum Entropy<br>[6] IP_HCC_lbp.3D.m1_glszm_Zone Entropy<br>[7] TP/DP_Liver_wavelet.LHH_gldm_Small Dependence High Gray Level Emphasis<br>[8] OP_HCC_square_glszm_Zone Variance<br>[9] AP_Liver_wavelet.LHH_glrlm_Run Length Nonuniformity Normalized<br>[10] OP_HCC_exponential_glszm_Zone Variance<br>[11] OP_Liver_lbp.2D_glszm_Zone Variance<br>[12] T2WI_HCC_logarithm_glszm_Large Area Low Gray Level Emphasis<br>[13] AP_Liver_wavelet.LHH_gldm_Small Dependence High Gray Level Emphasis<br>[14] T2WI_HCC_wavelet.LLL_glrlm_Long Run Low Gray Level Emphasis<br>[15] OP_Liver_square_glszm_ZoneVariance<br>[16] AP_HCC_lbp.3D.m1_first order_90 Percentile<br>[17] OP_HCC_exponential_glszm_Size Zone Nonuniformity Normalized<br>[18] OP_HCC_lbp.3D.k_first order_Minimum<br>[19] OP_HCC_lbp.3D.m1_glszm_Size Zone Nonuniformity Normalized<br>[20] T2WI_Liver_wavelet.HLL_gldm_Dependence Nonuniformity Normalized | [1] T2WI_HCC_original_glrlm_Long Run Low Gray Level Emphasis<br>[2] PVP_Liver_wavelet.LHH_glcm_Sum Entropy<br>[3] IP_HCC_lbp.3D.m1_glszm_Zone Entropy<br>[4] TP/DP_Liver_wavelet.LHH_gldm_Small Dependence High Gray Level Emphasis<br>[5] OP_Liver_lbp.2D_glszm_Zone Variance<br>[6] AP_Liver_wavelet.LHH_gldm_Small Dependence High Gray Level Emphasis<br>[7] AP_HCC_lbp.3D.m1_first order_90 Percentile<br>[8] T2WI_Liver_wavelet.HLL_gldm_Dependence Nonuniformity Normalized | 1.563 (1.352, 1.807)<br>0.626 (0.515, 0.761)<br>1.353 (1.177, 1.554)<br>1.207 (0.934, 1.56)<br>1.159 (0.961, 1.398)<br>0.664 (0.486, 0.906)<br>1.422 (1.117, 1.81)<br>0.693 (0.571, 0.841) | <0.001<br><0.001<br><0.001<br>0.151<br>0.123<br>0.010<br>0.004<br><0.001 |
| HCC with 5 mm tumor border extension + liver | [1] OP_HCC.peri5mm_wavelet.HHL_glszm_Size Zone Nonuniformity Normalized<br>[2] OP_HCC.peri5mm_lbp.3D.m2_glszm_Gray Level Nonuniformity<br>[3] PVP_Liver_wavelet.HLH_glcm_Sum Entropy<br>[4] OP_HCC.peri5mm_square_glszm_Gray Level Nonuniformity<br>[5] OP_HCC.peri5mm_lbp.2D_glszm_Gray Level Nonuniformity<br>[6] IP_HCC.peri5mm_square_glszm_Gray Level Nonuniformity<br>[7] PVP_HCC.peri5mm_wavelet.LHH_glcm_Sum Entropy<br>[8] PVP_Liver_wavelet.LHH_glrlm_Run Length Nonuniformity Normalized<br>[9] AP_HCC.peri5mm_wavelet.HLH_glszm_Zone Entropy<br>[10] IP_HCC.peri5mm_wavelet.HHL_glszm_Zone Entropy<br>[11] OP_HCC.peri5mm_exponential_glszm_Size Zone Nonuniformity Normalized<br>[12] OP_HCC.peri5mm_lbp.3D.m1_glszm_Gray Level Nonuniformity<br>[13] PVP_HCC.peri5mm_wavelet.HHH_glszm_Zone Entropy<br>[14] AP_Liver_wavelet.LHH_gldm_Small Dependence Emphasis<br>[15] PVP_Liver_wavelet.LHH_glcm_Sum Entropy<br>[16] TP/DP_Liver_wavelet.LHH_gldm_Small Dependence High Gray Level Emphasis<br>[17] OP_HCC.peri5mm_lbp.2D_glszm_Size Zone Nonuniformity Normalized<br>[18] OP_HCC.peri5mm_wavelet.HHH_glszm_Size Zone Nonuniformity Normalized<br>[19] IP_HCC.peri5mm_wavelet.HHL_glszm_Size Zone Nonuniformity Normalized<br>[20] OP_Liver_lbp.3D.m1_glszm_Zone Variance | [1] OP_HCC.peri5mm_lbp.3D.m2_glszm_Gray Level Nonuniformity<br>[2] AP_HCC.peri5mm_wavelet.HLH_glszm_Zone Entropy<br>[3] IP_HCC.peri5mm_wavelet.HHL_glszm_Zone Entropy<br>[4] PVP_Liver_wavelet.LHH_glcm_Sum Entropy<br>[5] IP_HCC.peri5mm_wavelet.HHL_glszm_Size Zone Nonuniformity Normalized | 1.132 (1.003, 1.278)<br>1.325 (1.042, 1.686)<br>1.929 (1.391, 2.675)<br>0.640 (0.551, 0.744)<br>1.419 (1.033, 1.950) | 0.044<br>0.022<br><0.001<br><0.001<br>0.031 |
| HCC with 10 mm tumor border extension + liver | [1] OP_HCC.peri10mm_lbp.3D.m1_glszm_Zone Variance<br>[2] AP_Liver_wavelet.LHH_glrlm_Run Length Nonuniformity Normalized<br>[3] AP_HCC.peri10mm_wavelet.LHL_glszm_Zone Entropy<br>[4] AP_HCC.peri10mm_wavelet.LHH_glcm_Sum Entropy<br>[5] OP_Liver_lbp.2D_glszm_Zone Entropy<br>[6] IP_HCC.peri10mm_lbp.3D.m2_glszm_Zone Entropy<br>[7] IP_HCC.peri10mm_square_glszm_Size Zone Nonuniformity Normalized<br>[8] TP/DP_Liver_wavelet.LHH_gldm_Small Dependence High Gray Level Emphasis<br>[9] PVP_Liver_wavelet.LHH_glcm_Sum Entropy<br>[10] OP_HCC.peri10mm_wavelet.HHL_glszm_Zone Entropy<br>[11] TP/DP_Liver_wavelet.LHH_gldm_Small Dependence Emphasis<br>[12] AP_Liver_wavelet.HLH_first order_Minimum<br>[13] OP_HCC.peri10mm_wavelet.HHL_glszm_Size Zone Nonuniformity Normalized<br>[14] TP/DP_Liver_wavelet.LHH_glcm_Sum Entropy<br>[15] T2WI_HCC.peri10mm_wavelet.LHL_glrlm_Short Run High Gray Level Emphasis<br>[16] OP_Liver_lbp.3D.m1_glszm_Zone Variance<br>[17] OP_Liver_exponential_glszm_Zone Variance<br>[18] PVP_HCC.peri10mm_wavelet.HLL_glrlm_Long Run Emphasis<br>[19] OP_HCC.peri10mm_lbp.2D_glszm_Zone Variance<br>[20] OP_Liver_gradient_glszm_Zone Entropy | [1] OP_HCC.peri10mm_lbp.3D.m1_glszm_Zone Variance<br>[2] IP_HCC.peri10mm_lbp.3D.m2_glszm_Zone Entropy<br>[3] IP_HCC.peri10mm_square_glszm_Size Zone Nonuniformity Normalized<br>[4] PVP_Liver_wavelet.LHH_glcm_Sum Entropy<br>[5] OP_HCC.peri10mm_wavelet.HHL_glszm_Zone Entropy<br>[6] AP_Liver_wavelet.HLH_first order_Minimum<br>[7] T2WI_HCC.peri10mm_wavelet.LHL_glrlm_Short Run High Gray Level Emphasis | 1.160 (1.009, 1.335)<br>0.276 (0.078, 0.972)<br>0.260 (0.075, 0.902)<br>0.614 (0.521, 0.723)<br>1.478 (1.191, 1.834)<br>1.490 (1.131, 1.961)<br>0.685 (0.543, 0.865) | 0.038<br>0.045<br>0.034<br><0.001<br><0.001<br>0.005<br>0.001 |
| HCC with 20 mm tumor border extension + liver | [1] AP_Liver_wavelet.LHH_gldm_Small Dependence High Gray Level Emphasis<br>[2] PVP_Liver_wavelet.LHH_glrlm_Run Length Nonuniformity Normalized<br>[3] PVP_Liver_wavelet.LHH_gldm_Small Dependence Emphasis<br>[4] PVP_Liver_wavelet.LHH_gldm_Small Dependence High Gray Level Emphasis<br>[5] OP_Liver_lbp.2D_glszm_Zone Variance<br>[6] TP/DP_Liver_wavelet.LHH_gldm_Small Dependence Emphasis<br>[7] TP/DP_Liver_wavelet.LHH_glcm_Sum Entropy<br>[8] PVP_Liver_wavelet.LHH_glcm_Sum Entropy<br>[9] T2WI_HCC.peri20mm_wavelet.HLL_glrlm_Short Run Low Gray Level Emphasis<br>[10] OP_Liver_exponential_glszm_Zone Variance<br>[11] AP_Liver_wavelet.LHH_glcm_Sum Entropy<br>[12] T2WI_HCC.peri20mm_wavelet.HLH_glszm_Size Zone Nonuniformity<br>[13] AP_Liver_wavelet.HLH_first order_Range<br>[14] T2WI_Liver_wavelet.HLL_gldm_Dependence Nonuniformity Normalized<br>[15] OP_Liver_gradient_glszm_Zone Variance<br>[16] TP/DP_HCC.peri20mm_wavelet.HLL_glcm_Sum Squares<br>[17] AP_Liver_wavelet.LHH_glrlm_Run Length Nonuniformity Normalized<br>[18] PVP_HCC.peri20mm_wavelet.HLL_first order_Uniformity<br>[19] TP/DP_Liver_square_glszm_Size Zone Nonuniformity<br>[20] PVP_HCC.peri20mm_wavelet.HLL_gldm_Gray Level Variance | [1] PVP_Liver_wavelet.LHH_glrlm_Run Length Nonuniformity Normalized<br>[2] PVP_Liver_wavelet.LHH_gldm_Small Dependence Emphasis<br>[3] OP_Liver_lbp.2D_glszm_Zone Variance<br>[4] T2WI_HCC.peri20mm_wavelet.HLL_glrlm_Short Run Low Gray Level Emphasis<br>[5] AP_Liver_wavelet.LHH_glcm_Sum Entropy (AP)<br>[6] AP_Liver_wavelet.HLH_first order_Range (AP)<br>[7] T2WI_Liver_wavelet.HLL_gldm_Dependence Nonuniformity Normalized<br>[8] PVP_HCC.peri20mm_wavelet.HLL_first order_Uniformity<br>[9] TP/DP_Liver_square_glszm_Size Zone Nonuniformity | 1.877 (1.149, 3.065)<br>0.661 (0.438, 0.996)<br>1.173 (0.974, 1.414)<br>0.606 (0.471, 0.778)<br>0.789 (0.602, 1.035)<br>0.631 (0.460, 0.864)<br>0.837 (0.682, 1.026)<br>0.326 (0.136, 0.782)<br>1.311 (1.114, 1.543) | 0.012<br>0.048<br>0.093<br><0.001<br>0.087<br>0.004<br>0.087<br>0.012<br>0.001 |

Data in parentheses are 95% confidence intervals.

AP, arterial phase; DP, delayed phase; HCC; hepatocellular carcinoma; IP, in phase; OP, opposed phase; PVP, portal venous phase; TP, transitional phase; T2WI, T2-weighted imaging.

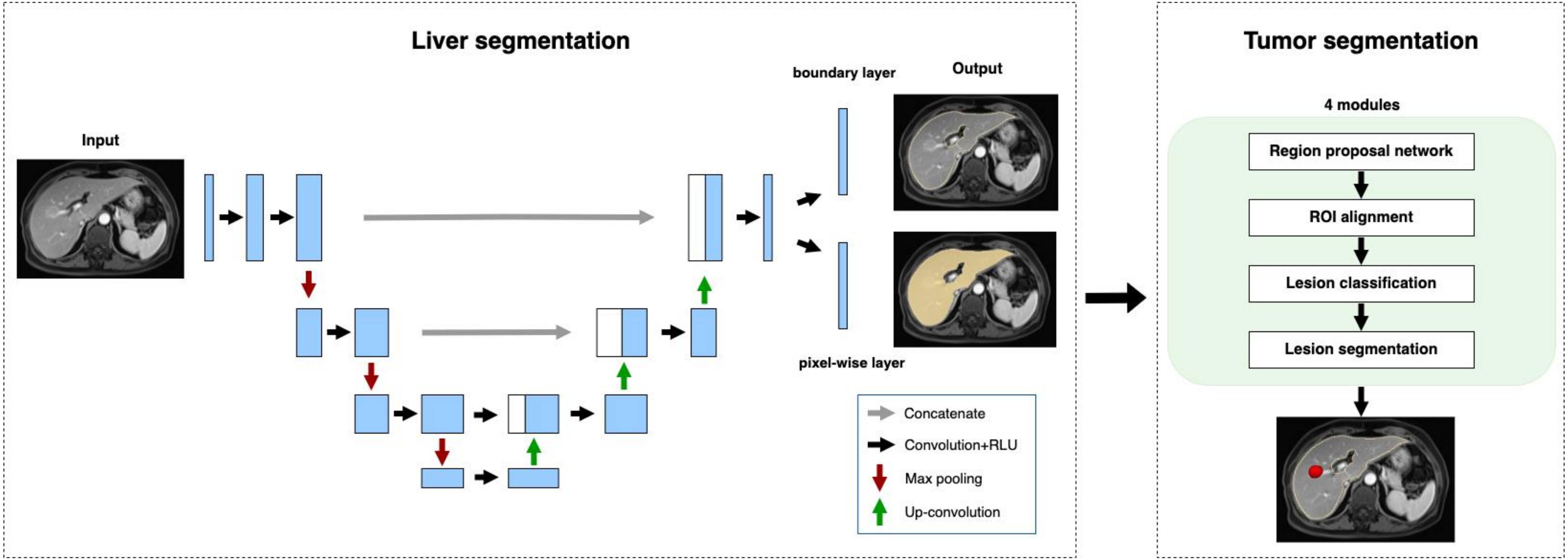


**Figure S1** 3D-Unet Architectures of Liver and Tumor Segmentation Models. RLU = rectified linear unit, ROI = region of interest.

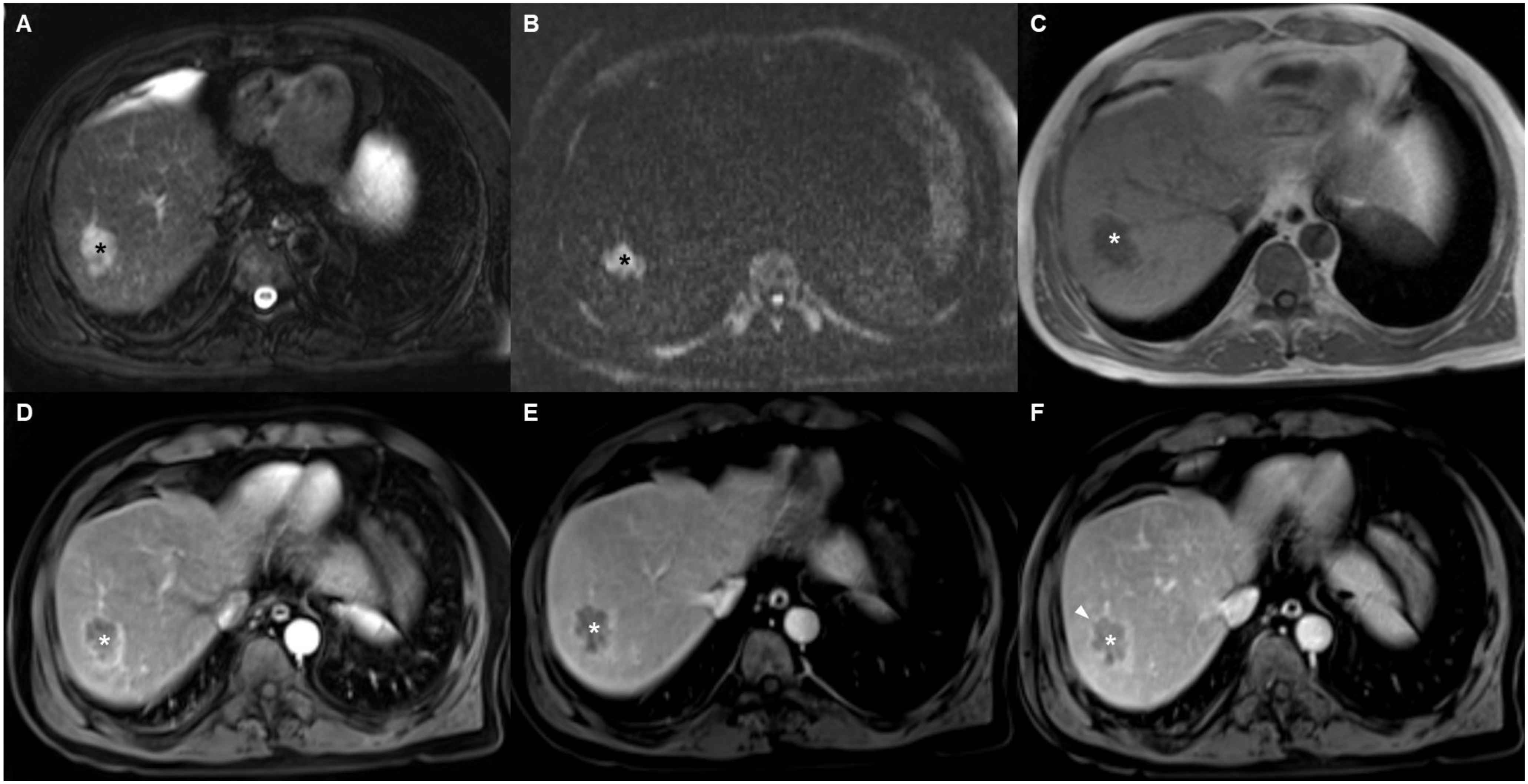


**Figure S2** Representative Images of A Patient with HCC at High Risk of Early Recurrence Determined by the Hybrid Model. Pathologically confirmed moderately differentiated HCC in a 50-year-old man without microvascular invasion. MRI with extracellular contrast agent demonstrated a 5.2 cm HCC in liver segment VII. The tumor (*****) showed mild to moderate hyperintensity on (**A**) T2-weighted image, diffusion restriction on (**B**) diffusion-weighted image (b = 1500 s/mm$^2$), hypointensity on (**C**) in phase image, rim APHE on (**D**) late arterial phase image, non-smooth tumor margin on (**E**) portal venous phase image, and incomplete tumor “capsule” (**arrowhead**) on (**F**) delayed phase image. This patient had two risk factors (rim APHE and incomplete tumor “capsule”) for early recurrence, with the radiomic score of -0.11 points. The final calculated score according to the hybrid model was 1.67 points, corresponding to the high-risk group (≥1.25 points). Early recurrence occurred after a follow-up period of 8.7 months. APHE, arterial phase hyperenhancement; HCC, hepatocellular carcinoma; MRI, magnetic resonance imaging.
